# Pyronaridine–artesunate or dihydroartemisinin–piperaquine combined with single low-dose primaquine to prevent *Plasmodium falciparum* malaria transmission in Ouélessébougou, Mali: a four-arm, single-blind, phase 2/3, randomised trial

**DOI:** 10.1016/S2666-5247(21)00192-0

**Published:** 2022-01

**Authors:** William Stone, Almahamoudou Mahamar, Koualy Sanogo, Youssouf Sinaba, Sidi M Niambele, Adama Sacko, Sekouba Keita, Ahamadou Youssouf, Makonon Diallo, Harouna M Soumare, Harparkash Kaur, Kjerstin Lanke, Rob ter Heine, John Bradley, Djibrilla Issiaka, Halimatou Diawara, Sekou F Traore, Teun Bousema, Chris Drakeley, Alassane Dicko

**Affiliations:** a Department of Infection Biology, London School of Hygiene & Tropical Medicine, London, UK; b MRC International Statistics and Epidemiology Group, London School of Hygiene & Tropical Medicine, London, UK; c Malaria Research and Training Centre, Faculty of Pharmacy and Faculty of Medicine and Dentistry, University of Sciences Techniques and Technologies of Bamako, Bamako, Mali; d Department of Medical Microbiology and Radboud Center for Infectious Diseases, Radboud University Medical Center, University of Nijmegen, Nijmegen, Netherlands; e Department of Pharmacy and Radboud Center for Infectious Diseases, Radboud University Medical Center, University of Nijmegen, Nijmegen, Netherlands

## Abstract

**Background:**

Pyronaridine–artesunate is the most recently licensed artemisinin-based combination therapy. WHO has recommended that a single low dose of primaquine could be added to artemisinin-based combination therapies to reduce *Plasmodium falciparum* transmission in areas aiming for elimination of malaria or areas facing artemisinin resistance. We aimed to determine the efficacy of pyronaridine–artesunate and dihydroartemisinin–piperaquine with and without single low-dose primaquine for reducing gametocyte density and transmission to mosquitoes.

**Methods:**

We conducted a four-arm, single-blind, phase 2/3, randomised trial at the Ouélessébougou Clinical Research Unit of the Malaria Research and Training Centre of the University of Bamako (Bamako, Mali). Participants were aged 5–50 years, with asymptomatic *P falciparum* malaria mono-infection and gametocyte carriage on microscopy, haemoglobin density of 9·5 g/dL or higher, bodyweight less than 80 kg, and no use of antimalarial drugs over the past week. Participants were randomly assigned (1:1:1:1) to one of four treatment groups: pyronaridine–artesunate, pyronaridine–artesunate plus primaquine, dihydroartemisinin–piperaquine, or dihydroartemisinin–piperaquine plus primaquine. Treatment allocation was concealed to all study staff other than the trial pharmacist and treating physician. Dihydroartemisinin–piperaquine and pyronaridine–artesunate were administered as per manufacturer guidelines over 3 days; primaquine was administered as a single dose in oral solution according to bodyweight (0·25 mg/kg; in 1 kg bands). The primary endpoint was percentage reduction in mosquito infection rate (percentage of mosquitoes surviving to dissection that were infected with *P falciparum*) at 48 h after treatment compared with baseline (before treatment) in all treatment groups. Data were analysed per protocol. This trial is now complete, and is registered with ClinicalTrials.gov, NCT04049916.

**Findings:**

Between Sept 10 and Nov 19, 2019, 1044 patients were assessed for eligibility and 100 were enrolled and randomly assigned to one of the four treatment groups (n=25 per group). Before treatment, 66 (66%) of 100 participants were infectious to mosquitoes, with a median of 15·8% (IQR 5·4–31·9) of mosquitoes becoming infected. In individuals who were infectious before treatment, the median percentage reduction in mosquito infection rate 48 h after treatment was 100·0% (IQR 100·0 to 100·0) for individuals treated with pyronaridine–artesunate plus primaquine (n=18; p<0·0001) and dihydroartemisinin–piperaquine plus primaquine (n=15; p=0·0001), compared with −8·7% (−54·8 to 93·2) with pyronaridine–artesunate (n=17; p=0·88) and 50·4% (13·8 to 70·9) with dihydroartemisinin–piperaquine (n=16; p=0·13). There were no serious adverse events, and there were no significant differences between treatment groups at any point in the frequency of any adverse events (Fisher's exact test p=0·96) or adverse events related to study drugs (p=0·64). The most common adverse events were headaches (40 events in 32 [32%] of 100 participants), rhinitis (31 events in 30 [30%]), and respiratory infection (20 events in 20 [20%]).

**Interpretation:**

These data support the use of single low-dose primaquine as an effective supplement to dihydroartemisinin–piperaquine and pyronaridine–artesunate for blocking *P falciparum* transmission. The new pyronaridine–artesunate plus single low-dose primaquine combination is of immediate relevance to regions in which the containment of partial artemisinin and partner-drug resistance is a growing concern and in regions aiming to eliminate malaria.

**Funding:**

The Bill & Melinda Gates Foundation.

**Translations:**

For the French, Spanish and Swahilil translations of the abstract see Supplementary Materials section.

**Research in context**

**Evidence before this study**

We searched PubMed on Jan 21, 2021, with no date or language restrictions, for studies assessing treatment with pyronaridine–artesunate (using the search terms “Pyronaridine-artesunate” OR “Pyramax” OR “AS-PYR” with down selection based on gametocyte detection), or pyronaridine–artesunate plus primaquine ([“Pyronaridine-artesunate” OR “Pyramax” OR “AS-PYR”] AND “Primaquine”). Among the 16 clinical or randomised controlled trials (2010–18) that met the search criteria, three studies presented data from a single trial done in Kenya (2018), in which gametocytes in patient blood were quantified by molecular methods up to 14 days after pyronaridine–artesunate treatment (showing 13·9–19·7% prevalence at day 14). Further studies were identified in which gametocytes present before and after pyronaridine–artesunate treatment were quantified by microscopy. None of the identified studies determined gametocyte infectivity to mosquitoes at any time. Narrowing this search to include studies assessing pyronaridine–artesunate in combination with primaquine identified only five relevant clinical trials. Two of these trials were focused on pharmacokinetic interaction between pyronaridine–artesunate and primaquine in healthy volunteers. The three other studies determined the safety and efficacy of pyronaridine–artesunate plus primaquine for treatment of malaria infection: two using a single low dose of 15 mg, one using the 14-day dosing regimen of primaquine for *Plasmodium vivax* hypnozoiticidal therapy. All three studies assessed gametocyte density using microscopy, and none determined gametocyte transmissibility.

An additional search of PubMed was done on the same date for studies assessing plasmodium infectivity to mosquitoes with mosquito feeding assays after any artemisinin-based combination therapy (ACT) treatment. For this search we used the terms “Malaria” AND (“ACT” OR “Artemisinin” OR “Artesunate” OR “Dihydroartemisinin”) AND (“DMFA” OR “MFA” OR “Mosquito feeding” OR “Mosquito feeding assay”). We identified 12 studies reporting mosquito infection data after ACT treatment. Excluding studies without specific timepoint identification, three distinct trials measured mosquito infection up to day 14 after treatment with dihydroartemisinin–piperaquine or artemether–lumefantrine: one done in Kenya (with data reported in two publications) and two in Cambodia. In one Cambodian study, only three (6%) of 48 individuals were infectious to mosquitoes at baseline. No studies assessed mosquito infection after day 14 and no studies were identified that used mosquito feeding assays following pyronaridine–artesunate treatment.

**Added value of this study**

This is the first clinical trial assessing the malaria transmission-reducing efficacy of pyronaridine–artesunate treatment alone or in combination with single low-dose primaquine, which has been recommended by WHO. The results suggest that pyronaridine–artesunate treatment alone has little efficacy against gametocytes and fails to prevent malaria transmission to mosquitoes shortly after treatment, whereas pyronaridine–artesunate plus single low-dose primaquine is highly effective as a schizonticidal and transmission-blocking drug combination. In addition to these insights, the study design allowed side-by-side comparison of pyronaridine–artesunate and pyronaridine–artesunate plus single low-dose primaquine with a standard second-line ACT (dihydroartemisinin–piperaquine), alone and in combination with single low-dose primaquine. These data also provide the first direct evidence of continued transmission to mosquitoes more than 14 days after ACT treatment: some individuals treated with dihydroartemisinin–piperaquine or pyronaridine–artesunate without primaquine continued to infect mosquitoes for up to 28 days after treatment.

**Implications of all the available evidence**

The results of this study add to a growing pool of evidence supporting the use of single low-dose primaquine alongside ACT treatment for the immediate prevention of onward *Plasmodium falciparum* malaria transmission. Before this study, the transmission-reducing efficacy of pyronaridine–artesunate with or without single low-dose primaquine was unknown. Pyronaridine–artesunate is a new and therefore valuable alternative antimalarial therapy in areas under threat from resistance or partial resistance to current first-line drug combinations. The data on the effects of pyronaridine–artesunate alone provide a resource to policy makers considering treatment prioritisation. Our data on the efficacy of pyronaridine–artesunate and dihydroartemisinin–piperaquine with single low-dose primaquine support the WHO recommendation that ACTs be combined with single low-dose primaquine to rapidly clear gametocytes and prevent transmission in areas fighting the spread of antimalarial drug resistance or in areas aiming to eliminate malaria.

## Introduction

Gametocytes are the only *Plasmodium* life stage that can be transmitted to mosquitoes. Drugs are a key component of malaria control strategies and yet, outside of mass drug administration,^1^ the gametocytocidal component of treatment receives little attention. The addition of gametocytocidal drugs to first-line malaria treatments could benefit control efforts, expedite elimination, and slow the spread of parasite strains that are resistant to standard schizonticides.^2^

Pyronaridine–artesunate is a recommended treatment for uncomplicated *Plasmodium falciparum* malaria in all malaria endemic regions.^3^ As the most recently licensed artemisinin-based combination therapy (ACT), pyronaridine–artesunate is being adopted in areas where the efficacy of current first-line and second-line drugs is decreasing.^4^ ACT treatment failures are associated with higher gametocyte prevalence.^5^ Regardless of clinical treatment outcome, ACTs have low activity against mature, transmissible gametocytes.^6^ Patients treated with artemether–lumefantrine or dihydroartemisinin–piperaquine can still harbour infectious gametocytes and these can persist after treatment.^7^ The duration of gametocyte infectivity to mosquitoes following ACT treatment is so far poorly defined and has not been assessed beyond the first week after treatment. Primaquine has potent gametocytocidal activity, with studies showing that a single low dose of 0·25 mg/kg is sufficient to neutralise infectivity to mosquitoes within 48 h.^8^, ^9^ This dose is considered safe in individuals deficient in glucose-6-phosphate dehydrogenase (G6PD), who are at risk of transient haemolysis after treatment with oxidative compounds including primaquine.^10^ WHO currently recommends treatment with ACT and single low-dose primaquine without G6PD testing to prevent onward *P falciparum* transmission in areas of artemisinin drug resistance or areas aiming for malaria elimination.^11^ Given that data from Rwanda have confirmed the de novo emergence of kelch13 mutations and associated delayed clearance of parasites,^12^, ^13^ novel ACTs and transmission-reducing measures, such as single low-dose primaquine, could be important components of efforts to avert a public health emergency.^2^

The transmission-reducing efficacy of pyronaridine–artesunate and pyronaridine–artesunate with single low-dose primaquine have not yet been tested, and there are indications that pyronaridine–artesunate might inhibit primaquine metabolism.^14^ We aimed to assess the efficacy of pyronaridine–artesunate with and without single low-dose primaquine and of dihydroartemisinin–piperaquine with and without primaquine for reducing the transmission of *P falciparum* gametocytes, and to examine the long-term duration of gametocyte circulation and infectivity to mosquitoes after treatment.

## Methods

### Study design and participants

We conducted a four-arm, single-blind, phase 2/3, randomised controlled trial at the Ouélessébougou Clinical Research Unit of the Malaria Research and Training Centre of the University of Bamako (Bamako, Mali). Screening and recruitment were done in the town of Ouélessébougou and surrounding villages. Ethics approval was granted by the Ethics Committee of the Faculty of Medicine, Pharmacy, and Dentistry of the University of Science, Techniques, and Technologies of Bamako (Bamako, Mali), and the Research Ethics Committee of the London School of Hygiene & Tropical Medicine (London, UK). Before the commencement of screening, our study team of clinicians and technicians met with community leaders, village health workers, and heads of households from each village to explain the study and obtain approval to conduct the study. Village health workers then used a door-to-door approach to inform households of the date and location where consenting and screening would take place. Participants were invited to enrol into the trial if they met the following criteria: positive for *P falciparum* gametocytes by microscopy (ie, ≥1 gametocytes recorded in a thick film against 500 white blood cells, equating to ≥16 gametocytes per μL with a standard conversion of 8000 white blood cells per μL blood); haemoglobin density of 9·5 g/dL or higher; age 5–50 years; bodyweight less than 80 kg; no clinical signs of malaria, defined by fever (≥37·5°C); no signs of chronic or severe disease; no allergies to any of the study drugs; and no reported use of antimalarial drugs over the past week. G6PD status was not tested. Exclusion criteria were pregnancy, known allergy to study treatments, clinical signs of severe malaria or hepatic injury or renal impairment, history of liver disease or renal impairment, family history of congenital prolongation of the corrected QT (QTc) interval, current or previous treatment with drugs metabolised by the enzyme cytochrome P450 2D6 (CYP2D6) or known to extend the QTc interval, and blood transfusion in the past 90 days. Before screening and study enrolment, participants provided written informed consent (if they were aged ≥18 years), assent with written parental consent (12–17 years), or written parental consent (<12 years).

### Randomisation and masking

Participants were individually randomly assigned (1:1:1:1) in blocks of 12, to one of four treatment groups: pyronaridine–artesunate (Pyramax; Shin Poong Pharmaceutical, Seoul, South Korea), pyronaridine–artesunate plus single low-dose primaquine (primaquine 0·25 mg/kg; ACE Pharmaceuticals, Zeewolde, Netherlands), dihydroartemisinin–piperaquine (Eurartesim; Sigma Tau, Gaithersburg, MD, USA), or dihydroartemisinin–piperaquine with single low-dose primaquine. An independent statistician at the Malaria Research and Training Centre randomly generated the treatment assignment using Stata version 16, which was linked to participant identification number. The statistician prepared sealed, opaque envelopes with the participant identification number on the outside and treatment assignment inside, which were sent to the study pharmacist. The study pharmacist provided treatment according to the contained assignment; consequently they and the study physician were not masked to treatment assignment, but all other investigators and staff involved in assessing all laboratory outcomes were masked. Participants could ask the study physician which treatment they received at any time.

### Procedures

ACT treatments were administered over 3 days (days 0, 1, and 2) under the direct supervision of the trial pharmacist, as per manufacturer instructions (appendix 4 p 2). All treatments were provided with food to facilitate metabolism. Primaquine tablets (26·3 mg) were dissolved to a 1 mg/mL solution in distilled water and administered orally according to bodyweight at 0·25 mL/kg (in 1 kg weight bands, taking the central point of the band for calculation of the 0·25 mg/kg dose) with a fruit-flavoured masking fluid, as described previously.^8^ For example, an individual with bodyweight of 50 kg would receive 12·5 mL of 1 mg/mL primaquine solution for a final dose of 0·25 mg/kg. Primaquine was administered as a single dose immediately after the first dose of ACT. Individuals in the pyronaridine–artesunate and dihydroartemisinin–piperaquine treatment groups received the same volume of masking fluid without primaquine, to equalise conditions between groups and to provide participants the option of remaining masked to treatment allocation. Participants in all treatment groups were treated with a full course of dihydroartemisinin–piperaquine at day 21 of follow-up, to prevent re-infection.

Blood samples were taken for haemoglobin density measurement (HemoCue; AB Leo Diagnostics, Helsingborg, Sweden), thick film microscopy, and molecular analysis of gametocyte density before treatment (day 0) and on days 1 (haemoglobin only), 2, 7, 14, 21, 28, 35, 42, and 49 after treatment (appendix 4 p 3). Additional blood samples were taken for infectivity assessments on days 0, 2, and 7 in all groups. In the dihydroartemisinin–piperaquine and pyronaridine–artesunate groups, blood samples were also taken for infectivity assays at days 10, 14, and then at any subsequent sampling timepoints if either of the previous assays (starting at day 10) had resulted in infected mosquitoes.

For infectivity assessments, 75 insectary-reared *Anopheles gambiae* females were allowed to feed for 15–20 min on venous blood samples (Lithium Heparin VACUETTE tube; Greiner Bio-One, Kremsmünster, Austria) through a warmed glass membrane feeder system (Coelen Glastechniek, Weldaad, Netherlands).^15^ Surviving mosquitoes were dissected 7 days after feeding and their midguts were stained with a 1% mercurochrome solution. The number of oocysts in the lamina of the midgut was recorded by trained technicians.

Thick film microscopy was performed as described previously,^9^ with asexual stages counted against 200 white blood cells and gametocytes counted against 500 white blood cells. For molecular gametocyte quantification, EDTA (edetic acid) blood (EDTA VACUETTE tube; Greiner Bio-One, Kremsmünster, Austria) was aliquoted into RNA protect cell reagent (Qiagen, Hilden, Germany) and stored at −80°C until temperature-tracked shipment on dry ice to Radboud University Medical Center (Nijmegen, Netherlands) for molecular assays. Total nucleic acids were extracted using a MagNAPure LC automated extractor (Total Nucleic Acid Isolation Kit—High Performance; Roche Applied Science, Indianapolis, IN, USA). Male and female gametocytes were quantified in a multiplex reverse-transcriptase quantitative PCR (RT-qPCR) assay as described previously (appendix 4 p 4).^16^ Samples were classified as negative for a particular gametocyte sex if the RT-qPCR-quantified density of gametocytes of that sex was less than 0·01 gametocytes per μL (ie, one gametocyte per 100 μL of blood sample). Clinical and haematological assessments were made on all study visits, and adverse events were graded by the study clinician for severity (mild, moderate, or severe) and relatedness to study medication (unrelated or probably not, possibly, probably, or definitely related). A drop in haemoglobin concentration by 2 g/dL or more between visits to less than 7 g/dL was categorised as a haematological adverse event. An external data safety and monitoring committee was assembled before the trial and discussed safety data after 40 participants were enrolled and after the last participant to enrol finished their final follow-up visit.

### Outcomes

Mosquito infectivity was assessed at three levels: mean number of oocysts in a sample of mosquitoes (ie, oocyst intensity), the proportion of mosquitoes infected with any number of oocysts (ie, mosquito infection rate), and infectivity of the study participant to any number of mosquitoes (ie, infectious individuals). The primary outcome measure was the median percentage change in mosquito infection rate at day 2 (48 h after treatment) within each group compared with baseline (pretreatment), with the same measure at day 7 as a secondary outcome. Percentage change was reported as percentage reduction (with 100% as total reduction of transmission, and negative values as enhanced transmission) and presented with IQR. Other secondary transmission outcomes were mosquito infectivity (oocyst intensity, mosquito infection rate, and infectious individuals) at days 2, 7, and at subsequent timepoints in the dihydroartemisinin–piperaquine and pyronaridine–artesunate treatment groups (to measure duration of infectivity without single low-dose primaquine). Secondary gametocyte outcome measures for all timepoints were gametocyte density, prevalence, sex ratio (ie, proportion of gametocytes that were male), circulation time, and area under the curve (AUC) of density over time. Safety outcomes at all timepoints were haemoglobin density and the total number of adverse events (including haematological adverse events). Differences in all transmission, gametocyte, and safety outcomes were compared between treatment groups as secondary comparisons. Some secondary and exploratory measures were not included in this report because assays could not be completed due to COVID-19-related constraints; these were measures of histidine rich protein 2/3 (HRP2/3) density, parasite genotyping, and molecular measures of asexual parasite density.

### Statistical analysis

Sample size was informed by a previous trial at the same site and with the same outcomes.^9^ Earlier data from the same site showed that 79% of individuals infected at least one mosquito before treatment, and among those who infected at least one mosquito, a median of 24% of mosquitoes became infected,^8^ giving an estimated probability of mosquito infection of 0·79 × 0·24=0·190. Using a SD of 0·24 for the change in proportion of infected mosquitoes before and after treatment (estimated from the same data), with 25 participants per group, we would have 80% power to detect a 95% or greater reduction in infectivity (from 0·190 to 0·009) as significant at the 0·05 level. With 25 participants per group, we estimated we would have 80% power to detect an 80% or greater reduction in the proportion of infectious individuals after treatment (a secondary outcome) as significant at the 0·05 level. Therefore, recruitment continued until 100 participants were enrolled (25 individuals assigned to each treatment group). Sample sizes were designed to assess reduction in mosquito infectivity over time within groups, not between groups (appendix 4 p 5).

Mosquito infection data were analysed at timepoints after baseline only for those individuals who were infectious at baseline (ie, infecting at least one mosquito with any number of oocysts); appendix 4 (pp 6, 8) also includes summaries and analyses for all individuals regardless of baseline infectivity. The prevalence of gametocytes and infectious individuals were compared within and between treatment groups using generalised linear models (family: binomial, Z score, coefficient with 95% CI) or Fisher's exact tests. Absolute haemoglobin density and percentage change in haemoglobin density (relative to baseline) were compared using paired *t* tests (*t* score for difference compared with day 0), and two-way *t* tests (*t* score for difference between ACT matched treatment groups [dihydroartemisinin–piperaquine *vs* dihydroartemisinin–piperaquine plus primaquine, pyronaridine–artesunate *vs* pyronaridine–artesunate plus primaquine] at each timepoint). The proportion of gametocytes that were male was calculated for all values with total gametocyte densities of at least 0·2 gametocytes per μL.^9^ Gametocyte circulation time was calculated to determine the mean number of days that a mature gametocyte circulates in the blood before clearance, using a deterministic compartmental model that assumes a constant rate of clearance and has a random effect to account for repeated measures on individuals, as described previously.^17^ Difference in circulation time was analysed using *t* tests (*t* score for difference between ACT matched treatment groups). AUC of gametocyte density per participant over time was calculated using the linear trapezoid method using the first 28 days of observation only,^18^ and was analysed by fitting linear regression models to the log_10_ adjusted AUC values, with adjustment for baseline gametocyte density (*t* score, coefficient with 95% CI). All other analyses of quantitative data were performed using Wilcoxon sign rank tests (Z score for difference compared with matched values at day 0) and Wilcoxon rank sum tests (Z score for difference between ACT matched treatment groups at each timepoint). All comparisons were defined before study completion and analyses were not adjusted for multiple comparisons. For all analyses, the threshold for statistical significance was set at p<0·05. Statistical analyses were done using STATA version 16.0 and SAS version 9.4.

This trial is registered with ClinicalTrials.gov, NCT04049916.

### Role of the funding source

The funder of the study had no role in study design, data collection, data analysis, data interpretation, or writing of the report.

## Results

**Figure 1:**
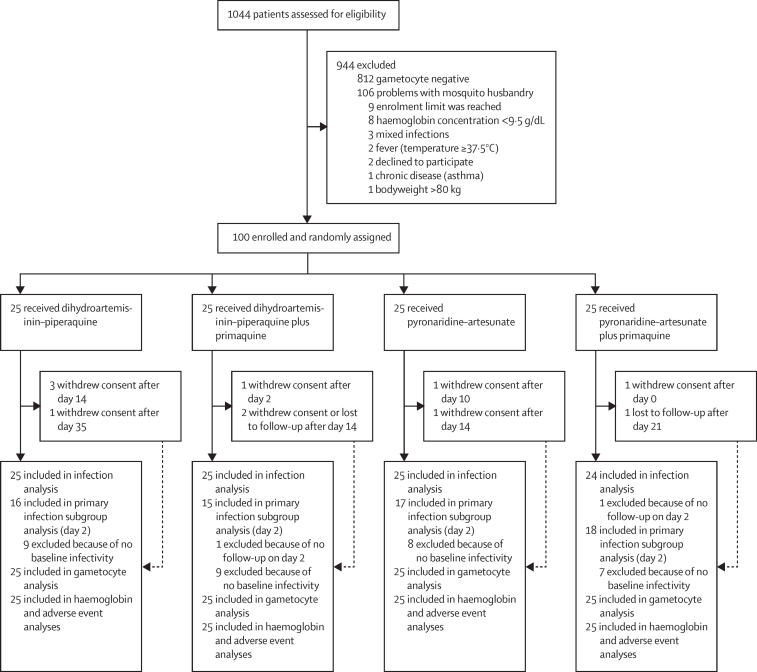
Trial profile

Between Sept 10 and Nov 19, 2019, 1044 patients were assessed for eligibility and 100 were enrolled and randomly assigned to one of the four treatment groups (n=25 per group; figure 1). 99 (99%) of 100 participants completed the day 2 study visit where the primary outcome measure was recorded, and 89 (89%) completed all visits to day 49. The final follow-up visit was completed on Jan 7, 2020. Participant characteristics were similar between treatment groups, although the proportion of male participants was lower in the dihydroartemisinin–piperaquine plus primaquine group than in the other treatment groups (table 1).

**Table 1:** Baseline characteristics

		**Dihydroartemisinin–piperaquine group (n=25)**	**Dihydroartemisinin–piperaquine plus primaquine group (n=25)**	**Pyronaridine–artesunate group (n=25)**	**Pyronaridine–artesunate plus primaquine group (n=25)**
Age, years		12 (9–16)	10 (8–16)	11 (9–14)	10 (7–12)
Sex					
	Female	12 (48%)	18 (72%)	9 (36%)	13 (52%)
	Male	13 (52%)	7 (28%)	16 (64%)	12 (48%)
Haemoglobin, g/dL		12·1 (11·1–12·9)	12·0 (11·0–12·4)	11·9 (11·4–12·8)	11·9 (11·0–13·2)
Gametocyte density by microscopy, parasites per μL		48 (32–80)	80 (48–192)	64 (32–96)	80 (32–144)
Gametocyte density by RT-qPCR,* parasites per μL		74·5 (37·6–126·4)	66·2 (22·9–189·9)	72·8 (26·1–121·7)	59·0 (25·8–146·1)
Asexual parasite prevalence by microscopy		22 (88%)	21 (85%)	13 (52%)	19 (76%)
Asexual parasite density by microscopy, parasites per μL		300 (120–2840)	400 (120–1400)	496 (200–1080)	1640 (400–8800)
Infectious to mosquitoes		16 (64%)	15 (60%)	17 (68%)	18 (72%)
Percentage of mosquitoes infected		23·6% (8·0–37·8)	22·9% (7·8–51·0)	6·2% (3·0–24·0)	9·9% (6·1–29·2)

Data are n (%) or median (IQR). RT-qPCR=reverse transcriptase quantitative PCR.

* qRT-PCR data are from individuals from whom baseline RNA samples were available: 25 in the dihydroartemisinin–piperaquine group, 24 in the dihydroartemisinin–piperaquine plus primaquine group, 23 in the pyronaridine–artesunate group, and 24 in the pyronaridine–artesunate plus primaquine group.

The median number of mosquitoes dissected in an individual mosquito feeding experiment was 65 (range 30–75). Before treatment, 66 (66%) of 100 participants were infectious to mosquitoes, with a median of 15·8% (IQR 5·4–31·9) of mosquitoes becoming infected (table 1).

**Figure 2:**
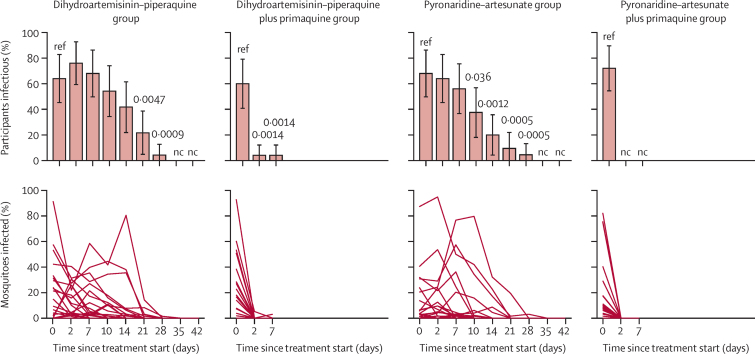
Participant infectivity to mosquitoes and percentage of mosquitoes infected in direct membrane feeding assays Error bars show 95% CI. Significant p values (<0·05) from generalised linear models (family: binary) testing differences within treatment groups with baseline as reference are shown. The denominator for participants infectious is the total number of participants still enrolled at the given timepoint, rather than the number tested for infectivity at that timepoint. Infectivity assays were discontinued when a participant did not infect any mosquitoes at two subsequent timepoints and were thereafter considered non-infectious. Full mosquito infection data including participants infectious with denominator as total participants tested are presented in appendix 4 (pp 6, 8). Each line in the plots showing percentage of mosquitoes infected represents an individual. Significant p values (<0·05) from Wilcoxon sign rank tests for differences in the median percentage of mosquitoes infected within treatment groups with baseline as reference are shown in appendix 4 (pp 6, 8). Timepoints not indicated on the x-axis were not tested. nc=not calculable.

At 48 h after treatment (day 2), 16 (64%) of 25 participants in the pyronaridine–artesunate group, 19 (76%) of 25 in the dihydroartemisinin–piperaquine group, none of 25 in the pyronaridine–artesunate plus primaquine group, and one (4%) of 24 in the dihydroartemisinin–piperaquine plus primaquine group infected any number of mosquitoes (figure 2, appendix 4 p 6). In individuals who were infectious before treatment, the median percentage reduction in mosquito infection rate 48 h after treatment was 100·0% (IQR 100·0 to 100·0) for individuals treated with pyronaridine–artesunate plus primaquine (n=18; p<0·0001) and dihydroartemisinin–piperaquine plus primaquine (n=15; p=0·0001), compared with −8·7% (−54·8 to 93·2) with pyronaridine–artesunate (n=17; p=0·88) and 50·4% (13·8 to 70·9) with dihydroartemisinin–piperaquine (n=16; p=0·13; table 2).

**Table 2:** Median percentage reduction in mosquito infection rate for individuals who were infectious to mosquitoes before treatment

	**Day 2**				**Day 7**			
	n	Median reduction (IQR)	p value*	p value†	n	Median reduction (IQR)	p value*	p value†
Dihydroartemisinin–piperaquine group	16	50·4% (13·8 to 70·9)	0·013	ref	16	34·2% (−11·8 to 88·1)	0·14	ref
Dihydroartemisinin–piperaquine plus primaquine group	15	100·0% (100·0 to 100·0)	0·0001	<0·0001	15	100·0% (100·0 to 100·0)	<0·0001	<0·0001
Pyronaridine–artesunate group	17	−8·7% (−54·8 to 93·2)	0·88	ref	17	38·9% (−10·6 to 100·0)	0·13	ref
Pyronaridine–artesunate plus primaquine group	18	100·0% (100·0 to 100·0)	<0·0001	<0·0001	17	100·0% (100·0 to 100·0)	<0·0001	0·0001

Median reduction is the median percentage reduction (relative to baseline) in mosquito infection rate at the given timepoints; positive values denote reductions in percentage of mosquitoes infected, negative values denote increases. All values are for individuals who were infectious to mosquitoes before treatment (ie, infected any number of mosquitoes). Full details of mosquito feeding assay outcomes are shown in appendix 4 (pp 6, 8).

* Within-group comparison by Wilcoxon signed rank test (day 0 as reference).

† Between artemisinin-based combination therapy matched group comparison (ie, dihydroartemisinin–piperaquine *vs* dihydroartemisinin–piperaquine plus primaquine, pyronaridine–artesunate *vs* pyronaridine–artesunate plus primaquine) by Wilcoxon rank-sum test.

At day 7, 14 (56%) of 25 participants in the pyronaridine–artesunate group, 17 (68%) of 25 in the dihydroartemisinin–piperaquine group, none of 24 in the pyronaridine–artesunate plus primaquine group, and one (4%) of 24 in the dihydroartemisinin–piperaquine plus primaquine group were infectious to mosquitoes (a different infectious individual in the dihydroartemisinin–piperaquine plus primaquine group on days 2 and 7; figure 2, appendix 4 p 6). Mosquito infection rate had increased in the dihydroartemisinin–piperaquine and pyronaridine–artesunate treatment groups by day 7 such that there was only a significant within-person reduction from baseline in mosquito infection rate in the pyronaridine–artesunate plus primaquine and dihydroartemisinin–piperaquine plus primaquine treatment groups (table 2). 13 (54%) of 24 participants in the dihydroartemisinin–piperaquine group and nine (38%) of 24 in the pyronaridine–artesunate group infected mosquitoes at day 10 after treatment, declining to one (4%) of 23 and one (5%) of 22 by day 28, respectively (appendix 4 p 8). Among those individuals tested, none infected mosquitoes at day 35 (of n=5 in the dihydroartemisinin–piperaquine group, n=2 in the pyronaridine–artesunate group) or 42 (of n=1 in the dihydroartemisinin–piperaquine group, n=1 in the pyronaridine–artesunate group; appendix 4 p 8). Comparing outcomes between ACT matched treatment groups (dihydroartemisinin–piperaquine *vs* dihydroartemisinin–piperaquine plus primaquine, pyronaridine–artesunate *vs* pyronaridine–artesunate plus primaquine), the addition of primaquine to either treatment resulted in significant reductions in infectivity to mosquitoes, grouped average mosquito infection rate, and oocyst density by day 2 after treatment (appendix 4 p 6).

**Figure 3:**
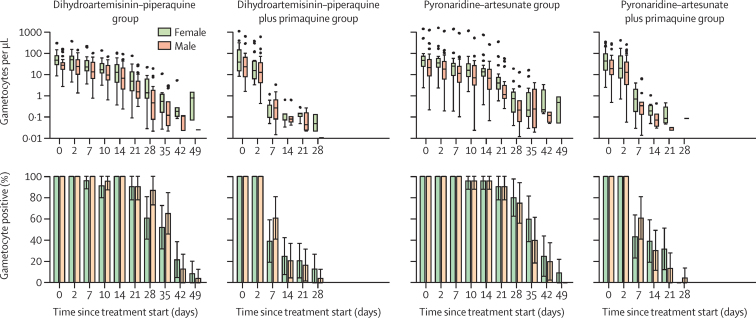
Gametocyte density and prevalence by gametocyte sex Error bars show 95% CI. On the gametocyte density plots, dots show outliers (1·5 times more than or less than the IQR). Boxplots show gametocyte densities for gametocyte-positive individuals only (ie, male or female density >0·01 gametocytes per μL). Tests for differences in gametocyte density (top row) were performed using Wilcoxon rank-sum tests and tests for differences in gametocyte prevalence (bottom row) were performed using Fisher's exact test. p values for female and male gametocytes are shown in appendix 4 (pp 9–11). Timepoints not indicated on the x-axis were not tested. Gametocyte circulation time, area under the curve, and other secondary outcomes are presented in appendix 4 (pp 9–11).

Gametocyte densities declined over time in all treatment groups, though much more rapidly in those who received primaquine, as reflected by the lower circulation time and AUC of male and female gametocytes (figure 3, appendix 4 pp 9–11). Within each group, gametocyte circulation time was similar for male and female gametocytes but AUC was lower for male gametocytes, reflecting the observed female bias in gametocyte density. Decreases in gametocyte density were preceded by a decrease in gametocyte infectivity at day 2 after primaquine treatment. Gametocyte sex ratios initially showed more female gametocytes in all groups (median proportion of male gametocytes 0·33 [IQR 0·21–0·49]), but showed significantly more male than female gametocytes in the dihydroartemisinin–piperaquine plus primaquine group by day 7 after treatment (appendix 4 pp 10, 12).

Mean haemoglobin density across all treatment groups 24 h after the start of treatment was 11·4 g/dL (range 8·7–15·2), representing a small but significant reduction in haemoglobin density relative to baseline in the dihydroartemisinin–piperaquine (mean change −3·88% [95% CI −6·96 to −0·81]), dihydroartemisinin–piperaquine plus primaquine (−6·04% [–8·52 to −3·56]), and pyronaridine–artesunate plus primaquine (−6·85% [–9·81 to −3·88]) treatment groups (appendix 4 pp 13–15). Haemoglobin density recovered to baseline levels by day 14 in the dihydroartemisinin–piperaquine and pyronaridine–artesunate plus primaquine groups, and by day 21 in the dihydroartemisinin–piperaquine plus primaquine group. At 24 h after treatment, there was a significant reduction in average haemoglobin density and percentage change in the pyronaridine–artesunate plus primaquine group compared with the pyronaridine–artesunate group (average density difference 0·76 g/dL [95% CI 0·02–1·50], average percentage change difference 4·44% [95% CI 0·30–8·59]); differences between ACT-matched treatment groups were not significant at any other timepoint.

Overall, 75 (75%) of 100 participants experienced a total of 173 adverse events during follow-up, 162 (94%) of which were categorised as mild severity and 11 (6%) as moderate (table 3). There was no difference between treatment groups in the proportion of participants who experienced an adverse event of mild (p=0·75) or moderate (p=0·90) severity (appendix 4 p 16). There were no serious adverse events, and there were no significant differences between treatment groups at any point in the frequency of any adverse events (p=0·96) or adverse events related to study drugs (p=0·64). The most common adverse events were headaches (40 events in 32 [32%] of 100 participants), rhinitis (31 events in 30 [30%]), and respiratory infection (20 events in 20 [20%]). No haematological adverse events occurred.

**Table 3:** Adverse events

	**Overall**		**Dihydroartemisinin–piperaquine group**		**Dihydroartemisinin–piperaquine plus primaquine group**		**Pyronaridine–artesunate group**		**Pyronaridine–artesunate plus primaquine group**	
	Adverse events	Adverse events linked to treatment	Adverse events	Adverse events linked to treatment	Adverse events	Adverse events linked to treatment	Adverse events	Adverse events linked to treatment	Adverse events	Adverse events linked to treatment
Cough	11 (0)	..	3 (0)	..	4 (0)	..	3 (0)	..	1 (0)	..
Diarrhoea	2 (0)	..	0 (0)	..	2 (0)	..	0 (0)	..	0 (0)	..
Fatigue	3 (2)	1 (0)	0 (0)	..	1 (1)	..	2 (1)	1 (0)	0 (0)	..
Gastroenteritis	1 (0)	..	0 (0)	..	1 (0)	..	0 (0)	..	0 (0)	..
Headaches	1 (0)	..	0 (0)	..	0 (0)	..	1 (0)	..	0 (0)	..
Myalgia	1 (0)	..	0 (0)	..	0 (0)	..	1 (0)	..	0 (0)	..
Nausea	16 (0)	11 (0)	6 (0)	4 (0)	2 (0)	1 (0)	4 (0)	3 (0)	4 (0)	3 (0)
Pruritus	4 (0)	2 (0)	2 (0)	1 (0)	0 (0)	..	1 (0)	1 (0)	1 (0)	..
Pyoderma	1 (0)	..	0 (0)	..	0 (0)	..	0 (0)	..	1 (0)	..
Respiratory Infection	20 (1)	..	5 (1)	..	4 (0)	..	6 (0)	..	5 (0)	..
Rhinitis	31 (0)	..	11 (0)	..	5 (0)	..	8 (0)	..	7 (0)	..
Vomiting	9 (0)	6 (0)	2 (0)	2 (0)	1 (0)	1 (0)	32 (0)	..	3 (0)	1 (0)
Wound	3 (0)	..	0 (0)	..	1 (0)	..	2 (0)	..	0 (0)	..
Abdominal pain	12 (1)	5 (0)	21 (0)	..	3 (0)	..	3 (0)	2 (0)	4 (1)	..
Back pain	1 (0)	..	0 (0)	..	0 (0)	..	1 (0)	..	0 (0)	..
Conjunctivitis	1 (0)	..	1 (0)	..	0 (0)	..	0 (0)	..	0 (0)	..
Dermatophytosis	1 (0)	..	0 (0)	..	1 (0)	..	0 (0)	..	0 (0)	..
Dizziness	4 (1)	1 (0)	1 (0)	..	1 (1)	..	0 (0)	..	2 (0)	1
Fever	1 (0)	..	0 (0)	..	0 (0)	..	1 (0)	..	0 (0)	..
Headache	40 (6)	9 (1)	8 (1)	8 (3)	6 (2)	2 (0)	16 (2)	2 (0)	10 (1)	2 (0)
Loss of appetite	5 (0)	..	1 (0)	..	0 (0)	..	2 (0)	..	2 (0)	..
Mumps	1 (0)	..	1 (0)	..	0 (0)	..	0 (0)	..	0 (0)	..
Scratch on right cheek	1 (0)	..	0 (0)	..	0 (0)	..	1 (0)	..	0 (0)	..
Tooth decay	1 (0)	..	1 (0)	..	0 (0)	..	0 (0)	..	0 (0)	..
Tooth pain	1 (0)	..	0 (0)	..	1 (0)	..	0 (0)	..	0 (0)	..
Traumatic red eye	1 (0)	..	0 (0)	..	0 (0)	..	1 (0)	..	0 (0)	..
Any adverse event	173 (11)	36* (1)	44 (2)	12 (1)	33 (4)	4 (0)	56 (3)	11 (0)	40 (2)	9 (0)
Mild adverse events	162	35	44	11	33	4	56	11	40	9
Moderate adverse events	11	1†	2	1	4	0	3	0	2	0

Data are number of moderate and mild adverse events (number of moderate adverse events). There were no serious adverse events. Empty cells indicate absence of adverse events linked to drug treatment. Adverse events linked to drug treatment were defined as possibly, probably, or definitely related to treatment.

* All 36 adverse events classified as linked to the study drug were categorised as expected.

† Headache.

## Discussion

This study showed that a single low dose of primaquine (0·25 mg/kg) added to either dihydroartemisinin–piperaquine or pyronaridine–artesunate resulted in total or near-total reduction in *P falciparum* transmission to mosquitoes by 48 h after treatment in asymptomatic gametocyte carriers in Mali. By contrast, *P falciparum* transmission to mosquitoes continued for up to 28 days after treatment with dihydroartemisinin–piperaquine or pyronaridine–artesunate alone. In line with these findings, gametocyte circulation time and AUC were significantly lower in the groups who received primaquine than in the non-primaquine groups; however, decreases in gametocyte density and changes in gametocyte sex ratio were preceded by the observed reduction in infectivity.

Pyronaridine–artesunate is being deployed as the first-line antimalarial in areas of west Africa and has the potential to replace current first-line ACTs facing the threat of antimalarial drug resistance.^19^, ^20^ A trial published in 2018 showed that pyronaridine–artesunate was non-inferior to the most widely used ACTs, artemether–lumefantrine and artesunate–amodiaquine, for the treatment of uncomplicated malaria in Mali,^19^ and trials are planned to ascertain the safety and efficacy of single-dose and standard pyronaridine–artesunate dose regimens for clearance of asymptomatic *P falciparum* infection (NCT03814616). ACTs differ in their efficacy against gametocytes, with considerably longer gametocyte persistence following dihydroartemisinin–piperaquine than after artemether–lumefantrine.^21^ Our data from highly sensitive sex-specific RT-qPCR assays show near identical gametocyte circulation times for individuals treated with dihydroartemisinin–piperaquine or pyronaridine–artesunate, tentatively supporting earlier indications that pyronaridine–artesunate might be inferior to artemether–lumefantrine for gametocyte clearance.^22^, ^23^ Gametocyte kinetics might not reflect gametocyte infectivity,^24^ and the effect of pyronaridine–artesunate on transmission to mosquitoes has not previously been studied. We assessed the infectivity of single infections up to 42 days after treatment, preventing re-infection by retreatment with dihydroartemisinin–piperaquine at day 21.

In efforts to prolong the lifespan of first-line antimalarials and stem the spread of emergent resistance, supplementing ACTs with low doses of primaquine (as recommended by WHO)^11^ or similar gametocytocidal treatments^25^, ^26^ is likely to become more common. In the current study, all treatments were well tolerated clinically, with no significant differences in haematological status between primaquine and non-primaquine groups after the first 24 h from treatment. Indications for antagonism of primaquine metabolism by pyronaridine, as might have been observed for *Plasmodium vivax* radical cure,^14^ were not observed for *P falciparum* gametocyte clearance or transmission blockade; pyronaridine–artesunate plus primaquine treatment resulted in total transmission reduction within 48 h, and dihydroartemisinin–piperaquine plus primaquine resulted in near-total reduction. As in previous studies,^8^, ^9^ our data show that decreased infectivity after primaquine treatment preceded significant changes in gametocyte density or sex ratio, supporting the hypothesis that early sterilisation by primaquine is due to effects on gametocyte viability rather than clearance of one or both sexes of gametocyctes.^27^ Moreover, this study provides a robust platform for the evaluation of other potential gametocytocidal compounds, such as tafenoquine, which might offer an extended period of gametocytocidal and sporontocidal activity.^25^

This study has several limitations. First, we recruited high-density gametocyte carriers, which allowed us to collect high-quality data on post-treatment transmissibility,^8^, ^9^ but does not represent the average gametocyte-infected individual. Our estimates of (long-term) persistence of transmissible gametocytes therefore reflect the effect of antimalarial drugs on transmission potential from a relative minority of highly infectious individuals. Second, treatment with dihydroartemisinin–piperaquine at day 21 in all study groups, to prevent re-infection, meant that gametocyte kinetics from day 28 onwards might be influenced by this second treatment, although the effect of dihydroartemisinin–piperaquine on mature gametocytes is evidently low. Third, our treatment strategy circumvented substantial barriers to the more widespread adoption of single low-dose primaquine in Africa: drug availability and ease of use. At present, primaquine tablets are available from a small number of suppliers, at a single concentration of 26·3 mg primaquine phosphate (equivalent to 15 mg primaquine base). Our approach, in which we dissolved the tablets in water, allows precise adherence to WHO recommendations with regard to dose,^11^ but it is conceivable that suboptimal primaquine doses would be used with more conventional dose targeting.^28^ Optimal dosing strategies will need to consider evidence that mg/kg dosing of primaquine results in reduced systemic exposure to primaquine in children,^29^ and that variations in primaquine metabolism due to mutations in the CYP2D6 enzyme might affect primaquine efficacy independently of age and weight.^30^ Finally, although we saw significant differences in transmission outcomes associated with different treatments in this trial, the public health relevance of these findings must be confirmed in community treatment trials with transmission endpoints. The effect of gametocytocidal drugs on community-wide transmission will differ with recommendations for use and must be considered in such trials. Combination with ACTs as part of standard treatment might result in only modest effects on wider transmission,^31^ whereas mass drug administration or a screen-and-treat approach, which remove more individuals from the infectious reservoir, would be likely to have greater effect. Practical and financial considerations will govern the deployment of such approaches.

Given the concerns about the spread of partial artemisinin and partner-drug resistance in southeast Asia, alternative effective antimalarial therapies are needed to ease drug pressure and maintain treatment efficacy. The emergence of de novo kelch13-mutation-associated artemisinin resistance in Africa also highlights the importance of expanding the arsenal of available antimalarial treatments.^11^, ^13^ Our findings provide further evidence that single low-dose primaquine is a safe and effective addition to ACTs, including pyronaridine–artesunate, for blocking *P falciparum* transmission.

## Data sharing

Anonymised data reported in the manuscript will be made available to investigators who provide a methodologically sound proposal to the corresponding author. The protocol is available upon request.

## Declaration of interests

We declare no competing interests.

## Supplementary Materials

French translation of the abstract

Spanish translation of the abstract

Swahilil translation of the abstract
