# Supplementary material for: Pyronaridine–artesunate or dihydroartemisinin–piperaquine combined with single low-dose primaquine to prevent Plasmodium falciparum malaria transmission in Ouélessébougou, Mali: a four-arm, single-blind, phase 2/3, randomised trial
Source: Lancet Microbe. 2022 Jan;3(1):e41–51. doi: 10.1016/S2666-5247(21)00192-0 (PMC8721154; doi:10.1016/S2666-5247(21)00192-0)
Supplement: French translation of the abstract [file mmc1.pdf]

THE LANCET
Microbe

Supplementary appendix 1

This translation in French was submitted by the authors and we reproduce it as supplied. It has not been peer reviewed. *The Lancet*'s editorial processes have only been applied to the original in English, which should serve as reference for this manuscript.

Supplement to: Stone W, Mahamar A, Sanogo K, et al. Pyronaridine–artesunate or dihydroartemisinin–piperaquine combined with single low-dose primaquine to prevent *Plasmodium falciparum* malaria transmission in Ouélessébougou, Mali: a four-arm, single-blind, phase 2/3, randomised trial. *Lancet Microbe* 2021; published online Oct 21. https://doi.org/10.1016/S2666-5247(21)00192-0.

Cette traduction en français a été proposée par les auteurs et nous l'avons reproduite telle quelle. Elle n'a pas été examinée par des pairs. Les processus éditoriaux du *Lancet* n'ont été appliqués qu'à l'original en anglais et c'est cette version qui doit servir de référence pour ce manuscrit.

Abstract – French translation

Contexte
L'association pyronaridine-artésunate est la combinaison thérapeutique à base d'artémisinine la plus récemment autorisée. L'OMS a recommandé qu'une faible dose de primaquine soit ajoutée aux combinaisons thérapeutiques à base d'artémisinine pour réduire la transmission de Plasmodium falciparum dans les zones visant l'élimination du paludisme ou les zones confrontées à une résistance à l'artémisinine. Nous avons cherché à déterminer l'efficacité de la pyronaridine-artésunate et de la dihydroartémisinine-pipéraquine avec et sans primaquine à faible dose unique pour réduire la densité des gamétocytes et la transmission aux moustiques.

Méthodes
Nous avons mené un essai randomisé de phase 2/3 en simple aveugle à quatre bras à l'Unité de Recherche Clinique de Ouélessébougou du Centre de Recherche et de Formation sur le Paludisme de l'Université de Bamako (Bamako, Mali). Les participants étaient âgés de 5 à 50 ans, avec une mono-infection palustre à P. falciparum asymptomatique et un portage de gamétocytes à l'examen microscopique, un taux d'hémoglobine de 9,5 g/dL ou plus, un poids corporel inférieur à 80 kg et aucune utilisation de médicaments antipaludiques les sept jours précédents. Les participants ont été randomisés (ratio 1:1:1:1) à l'un des quatre groupes de traitement : pyronaridine-artésunate, pyronaridine-artésunate plus primaquine, dihydroartémisinine-pipéraquine ou dihydroartémisinine-pipéraquine plus primaquine. L'attribution du traitement a été cachée à tout le personnel de l'étude autre que les pharmaciens de l'essai et le médecin traitant. La dihydroartémisinine-pipéraquine et la pyronaridine-artésunate ont été administrées conformément aux directives du fabricant sur 3 jours ; la primaquine a été administrée en dose unique en solution buvable en fonction du poids corporel (0,25 mg/kg ; en bandes de 1 kg). Le critère de jugement principal était le pourcentage de réduction du taux d'infection des moustiques (pourcentage de moustiques survivants à la dissection qui étaient infectés par P. falciparum) 48 h après le traitement par rapport à la valeur initiale (avant le traitement). Les données ont été analysées par protocole. Cet essai qui est maintenant terminé, avait été enregistré auprès de ClinicalTrials.gov, NCT04049916.

Résultats
Entre le 10 septembre et le 19 novembre 2019, 1044 patients ont été évalués pour l'éligibilité et 100 ont été inscrits et assignés au hasard à l'un des quatre groupes de traitement (n = 25 par groupe). Avant le traitement, 66 (66 %) des 100 participants étaient infectieux pour les moustiques, avec une médiane de 15,8 % (écart interquartile 5,4–31,9) de moustiques infectés. Chez les individus qui étaient infectieux avant le traitement, le pourcentage médian de réduction du taux d'infection des moustiques 48 h après le traitement était de 100,0% (écart interquartile 100,0 à 100,0) pour les individus traités par pyronaridine-artésunate plus primaquine (n = 18 ; p <0,0001) et dihydroartémisinine-pipéraquine plus primaquine (n=15 ; p=0,0001), comparativement à –8,7 % (-54,8 à 93,2) avec pyronaridine-artésunate (n=17 ; p =0,88) et 50,4 % (13,8 à 70,9) avec la dihydroartémisinine-pipéraquine (n=16 ; p=0,13). Il n'y a eu aucun événement indésirable grave, et il n'y avait pas de différence significative entre les groupes de traitement à aucun moment dans la fréquence des événements indésirables (test exact de Fisher p = 0,96) ou des événements indésirables liés aux médicaments à l'étude (p = 0,64). Les événements indésirables les plus fréquents étaient les maux de tête (40 événements sur 32 [32 %] des 100 participants), la rhinite (31 événements sur 30 [30 %]) et les infections respiratoires (20 événements sur 20 [20 %]).

Interprétation
Ces données supportent l'utilisation de la primaquine à dose faible unique comme complément efficace à la dihydroartémisinine-pipéraquine et à la pyronaridine-artésunate pour bloquer la transmission de P falciparum. La nouvelle combinaison pyronaridine-artésunate plus primaquine à faible dose est d'une pertinence immédiate pour les régions dans lesquelles la résistance partielle à l'artémisinine et de la résistance aux médicaments partenaires est une préoccupation croissante et celles visant à éliminer le paludisme.
