# Supplementary material for: Pyronaridine–artesunate or dihydroartemisinin–piperaquine combined with single low-dose primaquine to prevent Plasmodium falciparum malaria transmission in Ouélessébougou, Mali: a four-arm, single-blind, phase 2/3, randomised trial
Source: Lancet Microbe. 2022 Jan;3(1):e41–51. doi: 10.1016/S2666-5247(21)00192-0 (PMC8721154; doi:10.1016/S2666-5247(21)00192-0)
Supplement: Spanish translation of the abstract [file mmc2.pdf]

THE LANCET
Microbe

Supplementary appendix 2

This translation in Spanish was submitted by the authors and we reproduce it as supplied. It has not been peer reviewed. *The Lancet*'s editorial processes have only been applied to the original in English, which should serve as reference for this manuscript.

Supplement to: Stone W, Mahamar A, Sanogo K, et al. Pyronaridine–artesunate or dihydroartemisinin–piperaquine combined with single low-dose primaquine to prevent *Plasmodium falciparum* malaria transmission in Ouélessébougou, Mali: a four-arm, single-blind, phase 2/3, randomised trial. *Lancet Microbe* 2021; published online Oct 21. https://doi.org/10.1016/S2666-5247(21)00192-0.

Los autores nos proporcionaron esta traducción al español y la reproducimos tal como nos fue entregada. No la hemos revisado. Los procesos editoriales de *The Lancet* se han aplicado únicamente al original en inglés, que debe servir de referencia para este manuscrito.

Abstract – Spanish translation

Antecedentes
La pironaridina-artesunato es la terapia combinada basada en artemisinina autorizada más recientemente. La OMS ha recomendado que se podría añadir una única dosis baja de primaquina a los tratamientos combinados basados en la artemisinina para reducir la transmisión de Plasmodium falciparum en las zonas que aspiran a eliminar el paludismo o en las que se enfrentan a la resistencia a la artemisinina. El objetivo de este estudiofue determinar la eficacia de la pironaridina-artesunato y la dihidroartemisinina-piperaquina con y sin una dosis única de primaquina para reducir la densidad de gametocitos y la transmisión a los mosquitos.

Métodos
Se realizó un ensayo aleatorio de fase 2/3, a ciegas, de cuatro brazos, en la Unidad de Investigación Clínica de Ouélessébougou del Centro de Investigación y Formación sobre la Malaria de la Universidad de Bamako (Bamako, Malí). Los participantes tenían entre 5 y 50 años de edad, con monoinfección asintomática de paludismo por P falciparum y eran portadores de gametocitos por microscopía, densidad de hemoglobina de 9.5 g/dL o superior, peso corporal inferior a 80 kg y sin uso de medicamentos antipalúdicos en la última semana. Los participantes fueron asignados aleatoriamente (1:1:1:1) a uno de los cuatro grupos de tratamiento: pironaridina-artesunato, pironaridina-artesunato más primaquina, dihidroartemisinina-piperaquina o dihidroartemisinina-piperaquina más primaquina. La asignación del tratamiento se ocultó a todo el personal del estudio, salvo al farmacéutico del ensayo y al médico tratante. La dihidroartemisinina-piperaquina y la pironaridina-artesunato se administraron según las directrices del fabricante durante 3 días; la primaquina se administró como dosis única en solución oral según el peso corporal (0-25 mg/kg; en bandas de 1 kg). El criterio de valoración primario fue la reducción porcentual de la tasa de infección de los mosquitos (porcentaje de mosquitos que sobrevivieron hasta la disección y que estaban infectados por P falciparum) a las 48 horas del tratamiento en comparación con la línea de base (antes del tratamiento) en todos los grupos de tratamiento. Los datos se analizaron según el protocolo. Este ensayo ha finalizado y está registrado en ClinicalTrials.gov, NCT04049916.

Resultados
Entre el 10 de septiembre y el 19 de noviembre de 2019, se evaluó la elegibilidad de 1044 pacientes y se registraron 100, que fueron asignados aleatoriamente a uno de los cuatro grupos de tratamiento (n=25 por grupo). Antes del tratamiento, 66 (66%) de los 100 participantes fueron infecciosos para los mosquitos, con una mediana de 15.8% (IQR 5.4-31.9) de mosquitos infectados. En los individuos que fueron infecciosos antes del tratamiento, la mediana de reducción de la tasa de infección por mosquitos 48 h después del tratamiento fue del 100.0% (IQR 100.0 a 100.0) para los individuos tratados con pironaridina-artesunato más primaquina (n=18; p<0-0001) y dihidroartemisinina-piperaquina más primaquina (n=15; p=0-0001), en comparación con -8.7% (-54.8 a 93.2) con pironaridina-artesunato (n=17; p=0-88) y 50.4% (13.8 a 70.9) con dihidroartemisinina-piperaquina (n=16; p=0-13). No se produjeron acontecimientos adversos graves, y no hubo diferencias significativas entre los grupos de tratamiento en ningún momento en cuanto a la frecuencia de cualquier evento adverso (prueba exacta de Fisher p=0.96) o eventos adversos relacionados con los fármacos del estudio (p=0.64). Los eventos adversos más comunes fueron dolores de cabeza (40 eventos en 32 [32%] de 100 participantes), rinitis (31 eventos en 30 [30%]) e infección respiratoria (20 eventos en 20 [20%]).

Interpretación
Estos datos apoyan el uso de una dosis única de primaquina como complemento eficaz de la dihidroartemisina-piperaquina y de la pironaridina-artesunato para bloquear la transmisión de P falciparum. La nueva combinación de pironaridina-artesunato más dosis única de primaquina tiene una importancia inmediata para las regiones en las que la contención de la resistencia parcial a la artemisinina y a los medicamentos asociados es una preocupación creciente y en las regiones que aspiran a eliminar la malaria.
