# Supplementary material for: Pyronaridine–artesunate or dihydroartemisinin–piperaquine combined with single low-dose primaquine to prevent Plasmodium falciparum malaria transmission in Ouélessébougou, Mali: a four-arm, single-blind, phase 2/3, randomised trial
Source: Lancet Microbe. 2022 Jan;3(1):e41–51. doi: 10.1016/S2666-5247(21)00192-0 (PMC8721154; doi:10.1016/S2666-5247(21)00192-0)
Supplement: Swahilil translation of the abstract [file mmc3.pdf]

THE LANCET
Microbe

Supplementary appendix 3

This translation in Swahili was submitted by the authors and we reproduce it as supplied. It has not been peer reviewed. *The Lancet*'s editorial processes have only been applied to the original in English, which should serve as reference for this manuscript.

Supplement to: Stone W, Mahamar A, Sanogo K, et al. Pyronaridine–artesunate or dihydroartemisinin–piperaquine combined with single low-dose primaquine to prevent *Plasmodium falciparum* malaria transmission in Ouélessébougou, Mali: a four-arm, single-blind, phase 2/3, randomised trial. *Lancet Microbe* 2021; published online Oct 21. https://doi.org/10.1016/S2666-5247(21)00192-0.

Tafsiri hii katika Swahili iliwasilishwa na waandishi na tunatengeneza tena kama hutolewa. Haijapitiwa. Mchakato wa hariri wa Lancet Global Health umetumika tu kwa asili kwa Kiingereza, ambayo inapaswa kutumika kama kumbukumbu kwa muswada hii.

Abstract – Swahili translation

Utangulizi
Pyronaridine–artesunate ni dawa mseto iliyopata leseni kipindi cha hivi karibuni kabisa. Shirika la Afya Duniani limependekeza kuwa dozi ndogo ya mara moja ya primaquine inaweza ikaongezewa katika dawa mseto nyingine ili kupunguza kusambaa kwa Plasmodium falciparum katika maeneo yanayolenga kumaliza malaria au katika maeneo yanayokumbana na usugu wa artemisinin. Tulilenga kutambua uwezo wa pyronaridine–artesunate na dihydroartemisinin–piperaquine ikiwa au isipokua na dozi ndogo ya mara moja ya primaquine katika kupunguza wingi wa seli za vimelea na kuenea kwenda kwa mbu.

Njia

Tulifanya utafiti wenye afua 4, iliyofumbwa upande mmoja, awamu ya 2/3, ya utafiti wa jaribio la nasibu uliofanyikia kitengo cha tafiti za afya Ouélessébougou cha kituo cha mafunzo na tafiti za malaria katika Chuo Kikuu cha Bamako (Bamako, Mali). Washiriki walikuwa na umri wa miaka 5 hadi 50 wenye maamukizi ya mara moja ya malaria wa P falciparum lakini sio wagonjwa wa malaria na walio na seli za vimelea vinavyoonekana kwa darubini, wenye wingi wa damu wa 9·5 g/dL au zaidi, wenye uzito wa mwili chini ya kilo 80 na ambao hawajatumia dawa za malaria kwa zaidi ya wiki. Washiriki walipangwa kiholela (1:1:1:1) kuingia katika mojawapo ya makundi manne ya tiba: pyronaridine–artesunate, pyronaridine–artesunate pamoja na primaquine, dihydroartemisinin–piperaquine, au dihydroartemisinin–piperaquine pamoja na primaquine. Utoaji wa dawa ulisitishwa kwa wafanyakazi wote wa utafiti isipokua kwa mfamasia na daktari anayetibu wa utafiti. Dihydroartemisinin–piperaquine na pyronaridine–artesunate zilitolewa kulingana na muongozo wa mtengenezaji kwa siku 3; primaquine ilitolewa kama dozi ya mara moja ya maji kulingana na uzito wa mwili (0·25 mg/kg; kwa bendi za 1 kg). Matokeo ya msingi ilikuwa kupungua kwa asilimia ya kiasi cha uambukizo kwa mbu (asilimia ya mbu wanaobaki ili kupasuliwa ambao waliambukizwa na P falciparum) wakati wa saa ya 48 baada ya tiba ikilinganishwa na hali ya mwanzo (kabla ya tiba) katika makundi yote ya tiba. Taarifa zilichakatwa kulingana na muongozo. Utafiti huu wa majaribio umemalizika kwa sasa, na umeandikishwa ClinicalTrials.gov, NCT04049916.

Matokeo
Kipindi kati ya Septemba 10 na Novemba 19, 2019, wagonjwa 1044 walikaguliwa kufikia vigezo na 100 waliandikishwa na kupangwa kiholela kuwa katika moja ya makundi ya tiba (idadi ya 25 kwa kundi). Kabla ya tiba, washiriki 66 (66%) kati ya washiriki 100 walikuwa na maambukizi yanayoweza kuenezwa na mbu, kwa wastani wa 15·8% (IQR 5·4–31·9) ya mbu watakaokuja kuambukizwa. Miongoni mwa watu walioweza kuambukiza kabla ya tiba, wastani wa asilimia ya kupungua kwa kiasi cha maambukizi ya mbu saa ya 48 baada ya tiba ulikuwa 100·0% (IQR 100·0 to 100·0) kwa watu waliotibiwa na pyronaridine–artesunate pamoja na primaquine (n=18; p<0·0001) na dihydroartemisinin–piperaquine pamoja na primaquine (n=15; p=0·0001), ikilinganishwa na –8·7% (–54·8 to 93·2) kwa pyronaridine–artesunate (n=17; p=0·88) na 50·4% (13·8 to 70·9) kwa dihydroartemisinin–piperaquine (n=16; p=0·13). Hakukuwa na madhara yoyote makubwa, na hakukuwa na tofauti yoyote ya maana kati ya makundi ya tiba katika kipindi chochote cha kutokea kwa madhara yoyote (kipimo halisi cha Fisher's p=0·96) au madhara yaliyohusiana na dawa za utafiti (p=0·64). Madhara ya mara kwa mara yalikuwa maimivu ya kichwa (ilitokea mara 40 miongoni mwa washiriki 32 [32%] kati ya washiriki 100), kubanwa mafua (ilitokea mara 31 miongoni mwa washiriki [30%]), na uambukizo katika njia ya hewa/upumuaji (ilitokea mara 20 miongoni mwa washiriki 20 [20%]).

Tafsiri
Taarifa hizi zinaunga mkono matumizi ya dozi ndogo ya mara moja ya primaquine kama nyongeza yenye ubora kwa dihydroartemisinin– piperaquine na pyronaridine–artesunate kwa ajili ya kuzuia maambukizi ya P falciparum. Mseto mpya wa pyronaridine–artesunate pamoja na dozi ndogo ya mara moja ya primaquine ina umuhimu wa haraka katika maeneo ambapo jitihada za awali za kuzuia usugu wa artemisinin na dawa mwenza zinaongezeka na katika maeneo yanayolenga kumaliza malaria.
