# Supplementary material for: Pyronaridine–artesunate or dihydroartemisinin–piperaquine combined with single low-dose primaquine to prevent Plasmodium falciparum malaria transmission in Ouélessébougou, Mali: a four-arm, single-blind, phase 2/3, randomised trial
Source: Lancet Microbe. 2022 Jan;3(1):e41–51. doi: 10.1016/S2666-5247(21)00192-0 (PMC8721154; doi:10.1016/S2666-5247(21)00192-0)
Supplement: Supplementary appendix 4 [file mmc4.pdf]

THE LANCET
Microbe

Supplementary appendix 4

This appendix formed part of the original submission and has been peer reviewed. We post it as supplied by the authors.

Supplement to: Stone W, Mahamar A, Sanogo K, et al. Pyronaridine–artesunate or dihydroartemisinin–piperaquine combined with single low-dose primaquine to prevent *Plasmodium falciparum* malaria transmission in Ouélessébougou, Mali: a four-arm, single-blind, phase 2/3, randomised trial. *Lancet Microbe* 2021; published online Oct 21. https://doi.org/10.1016/S2666-5247(21)00192-0.

Appendix

Supplementary information 1. Schizonticidal antimalarial treatment dosing 2
Dihydroartemisinin-Piperaquine (DP) 2
Pyronaridine-Artesunate 2
Supplementary figure 1. Schematic representation of sample collection and analysis pipeline 3
Supplementary table 1. Primer sequences and qPCR conditions for PfMGET CCp4 assay 4
Supplementary information 2. Primary endpoint and sample size 5
Supplementary table 2. Infectivity to mosquitoes (≤7 days post treatment) 6
A. Infectious individuals 6
B. Mosquito infection (infection rate and oocyst intensity) 6
Supplementary table 3. Median percent reduction in mosquito infection rate for individuals infectious before treatment (with 95% CI) 7
Supplementary table 4. Infectivity to mosquitoes (PA and DP arms only, ≥10 days post treatment) 8
A. Infectious individuals 8
B. Mosquito infection (infection rate and oocyst intensity) 8
Supplementary table 5. Gametocyte circulation time and area under the curve 9
Supplementary table 6. Total gametocyte density, prevalence and sex ratio 10
Supplementary table 7. Female (CCP4) and male (PfMGET) gametocyte density and prevalence 11
Supplementary figure 2. Proportion of gametocytes that were male 12
Supplementary table 8. Haemoglobin density and density change over time 13
Supplementary figure 3. Percent change in haemoglobin density. 14
Supplementary figure 4. Absolute haemoglobin concentration 15
Supplementary table 9. Prevalence of adverse events 16
References 17

Supplementary information 1. Schizonticidal antimalarial treatment dosing

A. Dihydroartemisinin-Piperaquine (DP)

Participants in the DP or DP-PQ arm were treated with standard doses of DP. DP treatment Tablets containing 160/320 mg piperaquine w. 20/40 mg dihydroartemisinin tablets (Eurartesim, Sigma Tau) were administered according to weight as per manufacturer guidelines shown below:

**Body weight (kg)**	**Total daily dose (mg)** (1x/day for 3 days)		**Tablet strength and number of tablets per dose**
	Piperaquine	DHA	
5 to <7	80	10	½ x 160mg / 20mg
7 to <13	160	20	1 x 160mg / 20mg
13 to <24	320	40	1 x 320mg / 40mg
24 to <36	640	80	2 x 320mg / 40mg
36 to <75	960	120	3 x 320mg / 40mg
75 to 80	1,280	160	4 x 320mg / 40mg
>80	Not eligible		

Participants in the DP and PA arms received a full course of DP at baseline and a second full course at day 21, to ensure prophylaxis for the duration of follow-up.

B. Pyronaridine-Artesunate

Participants in the PA or PA-PQ arm were treated with standard doses of PA. PA granules containing 60 mg pyronaridine-tetraphosphate/20mg artesunate (Pyramax, Shin Poong Pharmaceutical Co.) were administered to children <20kg, and PA tablets containing 180 mg pyronaridine-tetraphosphate/60mg artesunate were administered to children and adults >20kg, according to weight as per manufacturer guidelines shown below:

**Granules (Children <20kg)**			
**Body weight (kg)**	**Total daily dose (mg)** (1x/day for 3 days)		**Sachet strength and number of tablets per dose**
	Pyronaridine-tetraphosphate	*Artesunate*	
5 - <8kg	60	20	1 x 60mg/20mg
8 - <15kg	120	40	2 x 60mg/20mg
15 - <20kg	180	60	3 x 60mg/20mg

**Tablets (Children and adults >20kg)**			
**Body weight (kg)**	**Total daily dose (mg)** (1x/day for 3 days)		**Tablet strength and number of tablets per dose**
	Pyronaridine-tetraphosphate	*Artesunate*	
20 - <24kg	180	60	1 x 180mg/60mg
24 - <45kg	360	120	2 x 180mg/60mg
45-<65kg	540	180	3 x 180mg/60mg
>65kg	720	240	4 x 180mg/60mg

Supplementary figure 1. Schematic representation of sample collection and analysis pipeline

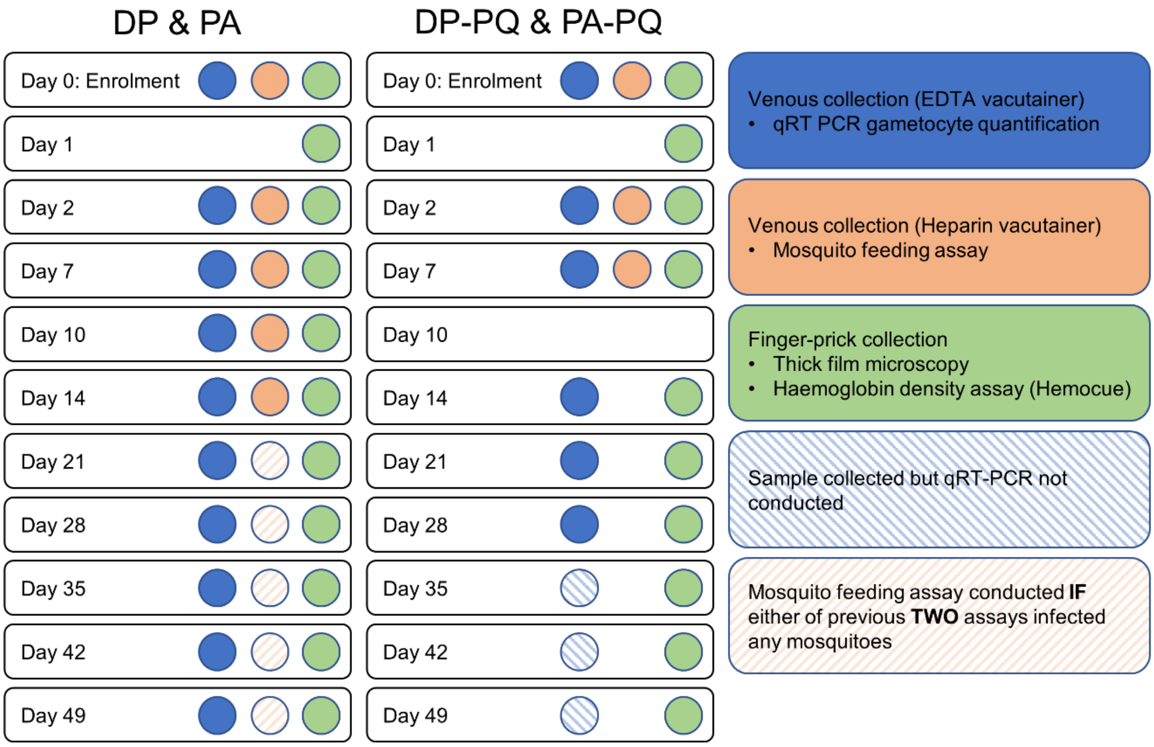

Supplementary table 1. Primer sequences and qPCR conditions for PfMGET CCp4 assay

PfMGET Primer/Probe Sequences

**Primers**	**Sequence**
Primer-FW (5'-3')	CGGTCCAAATATAAAAATCCTG
Primer-RV (5'-3')	TGTG TAACG TATG ATTCATTTTC
Probe (5'-3')	FAM-CAGCTCCAG CATTAAAACAC-BHQ1

CCp4 Primer/Probe Sequences

**Primers**	**Sequence**
Primer-FW (5'-3')	CACATGAATATGAGAATAAAATTG
Primer-RV (5'-3')	TAGGCGAACATGTGGAAAG
Probe (5'-3')	TexasRed-AGCAACAACGGTATGTGCCTTAAAACG-BHQ2

Male and female gametocyte quantification was performed as described previously, using a multiplex RT-qPCR assay (1). Assays were run using commercial RT-qPCR mixes (Luna® Universal Probe One-Step RT-qPCR Kit, New England Biolabs, Ipswich, MA, USA). FW = Forward primer. RV = Reverse primer.

Supplementary information 2. Primary endpoint and sample size

The primary endpoint of the current trial is the median within person percent reduction in mosquito infection rate (the percentage of mosquitoes surviving to dissection that each participant infected) at day 2 compared to baseline. Sample size was informed by previous trials (2, 3). In those studies, ~79% of individuals infected at least one mosquito before treatment, and among those who infected at least one mosquito, an average of 24% of mosquitoes became infected, giving an estimated probability of mosquito infection of $0{\cdot}79 \times 0{\cdot}24 = 0{\cdot}190$. Using a standard deviation of 0·24 for the change in proportion of infected mosquitoes before and after treatment (estimated from the same data), with 25 participants per arm, we would have 80% power to detect a 95% or greater reduction in infectivity (from 0·190 to 0·009) as statistically significant at the 0.05 level. With 25 participants per group, we would have 80% power to detect an 80% or greater reduction in the proportion of infectious individuals after treatment (a secondary outcome) as significant at the 0.05 level. Sample sizes were designed to assess reduction in mosquito infectivity over time within arms, not between arms. A formal comparison between arms would have necessitated larger sample sizes that affect the logistic feasibility of the study by the need to find sufficient gametocyte carriers in a single transmission season, and the required logistical intensity of mosquito membrane feeding assays (in terms of mosquito husbandry and labour). As in previous studies, we included between arm comparisons as secondary outcomes and report these data.

Supplementary table 2. Infectivity to mosquitoes (≤7 days post treatment)

A. Infectious individuals

Day of follow-up	Treatment arm	Infectious individuals* % (n/N)	p-value¥	p-value†
Day 0	Overall	66 (66/100)	-	-
	DP	64 (16/25)	*ref*	*ref*
	DP-PQ	60 (15/25)	*ref*	0.77
	PA	68 (17/25)	*ref*	*ref*
	PA-PQ	72 (18/25)	*ref*	0.76
Day 2	DP	76 (19/25)	0.36	*ref*
	DP-PQ	4.17 (1/24)	0.0014	0.0002
	PA	64 (16/25)	0.77	*ref*
	PA-PQ	0 (0/25)	*nc*	*nc*
Day 7	DP	68 (17/25)	0.77	*ref*
	DP-PQ	4.17 (1/24)	0.0014	0.0005
	PA	56 (14/25)	0.39	*ref*
	PA-PQ	0 (0/24)	*nc*	*nc*

B. Mosquito infection (infection rate and oocyst intensity)

		i. Infectious individuals						*ii. All individuals*					
Day of follow-up	Treatment arm	Average percent infection** Median % (IQR)	p-value¥	p-value†	Oocyst density Median (IQR)***	p-value¥	p-value†	Average percent infection** Median % (IQR)	p-value¥	p-value†	Oocyst density Median (IQR)****	p-value¥	p-value†
Day 0	*Overall*	15.77 (5.41-31.94)	-	-	0.24 (0.07-0.68)	-	-	4.73 (0-23.59)	-	-	0.06 (0-0.47)	-	-
	DP	23.59 (7.95-37.78)	*ref*	*ref*	0.47 (0.1-1.01)	*ref*	*ref*	6.56 (0-29.31)	*ref*	*ref*	0.07 (0-0.5)	*ref*	*ref*
	DP-PQ	22.86 (7.81-50.98)	*ref*	0.91	0.33 (0.08-2.57)	*ref*	0.72	7.35 (0-27.12)	*ref*	0.85	0.07 (0-0.63)	*ref*	0.94
	PA	6.15 (2.99-24)	*ref*	*ref*	0.08 (0.03-0.49)	*ref*	*ref*	2.99 (0-13.73)	*ref*	*ref*	0.03 (0-0.32)	*ref*	*ref*
	PA-PQ	9.89 (6.06-29.17)	*ref*	0.27	0.15 (0.07-0.57)	*ref*	0.29	7.14 (0-17.33)	*ref*	0.37	0.08 (0-0.27)	*ref*	0.38
Day 2	DP	13.59 (3.45-25.81)	0.0052	*ref*	0.27 (0.03-0.86)	0.036	*ref*	4 (1.33-21.88)	0.028	*ref*	0.04 (0.02-0.5)	0.13	*ref*
	DP-PQ	0 (0-0)	0.0007	<0.0001	0 (0-0)	0.0007	<0.0001	0 (0-0)	0.0002	<0.0001	0 (0-0)	0.0002	<0.0001
	PA	9.46 (1.64-23.44)	0.52	*ref*	0.1 (0.02-0.4)	0.44	*ref*	4 (0-12.5)	0.34	*ref*	0.04 (0-0.21)	0.30	*ref*
	PA-PQ	0 (0-0)	0.0002	<0.0001	0 (0-0)	0.0002	<0.0001	0 (0-0)	0.0001	<0.0001	0 (0-0)	0.0001	<0.0001
Day 7	DP	6.51 (2.06-27.78)	0.017	*ref*	0.07 (0.03-0.43)	0.0061	*ref*	3.03 (0-17.19)	0.14	*ref*	0.03 (0-0.27)	0.051	*ref*
	DP-PQ	0 (0-0)	0.0007	<0.0001	0 (0-0)	0.0007	<0.0001	0 (0-0)	0.0004	<0.0001	0 (0-0)	0.0004	<0.0001
	PA	2.5 (0-24.66)	0.19	*ref*	0.03 (0-0.37)	0.068	*ref*	1.52 (0-4.41)	0.48	*ref*	0.02 (0-0.04)	0.21	*ref*
	PA-PQ	0 (0-0)	0.0003	0.0001	0 (0-0)	0.0003	0.0001	0 (0-0)	0.0001	<0.0001	0 (0-0)	0.0001	<0.0001

A. Percentage of infectious individuals. *Individuals were classed as infectious if direct membrane feeding assays (DMFA) resulted in at least one mosquito with any number of oocysts. **B.** Mosquito infection measures are presented for i. all participants who were infectious at baseline, and ii. all participants regardless of baseline infectivity. **Average percent infection = Median percentage of mosquitoes infected by each participant. Each participant's mosquito infection rate was calculated as the number of mosquitoes infected as a percentage of all mosquitoes surviving to dissection. Oocyst densities are the median of all individuals' average oocyst densities. *nc* = not calculable, no positive observations. - = not tested. P-value¥ = Within group comparison. P-value† = Between ACT matched group comparison (i.e. DP with DP-PQ, PA with PA-PQ). *ref* = reference group.

Supplementary table 3. Median percent reduction in mosquito infection rate for individuals infectious before treatment (with 95% CI)

	Day 2			Day 7		
	n	Median % reduction (95% CI)	p-value	n	Median % reduction (95% CI)	p-value¥
DP	16	50.44 (26.35-74.54)	0.013¥	16	34.20 (-6.76-75.15)	0.141¥
DP-PQ	15	100 (*nc*)	0.0001¥	15	100 (*nc*)	<0.0001¥
Difference	-	*-49.56 (-75.49--23.62)*	*<0.0001†*	-	*-65.80 (-112.83--18.77)*	*<0.0001†*
PA	17	-8.67 (-73.22-55.87)	0.88¥	17	38.94 (-19.99-97.87)	0.1328¥
PA-PQ	18	100 (*nc*)	<0.0001¥	17	100 (*nc*)	<0.0001¥
Difference	-	*-108.67 (-178.01--39.34)*	*<0.0001†*	-	*-61.06 (-121.38--0.74)*	*0.0001†*

Median % reduction is the median percent reduction (relative to baseline) in mosquito infection rate at the given timepoints. Positive values denote reductions in percent infected, negative values denote increases. 95% Confidence intervals (CI) were calculated by bootstrapping with 1000 repetitions. All values are for individuals who were infectious to mosquitoes before treatment (i.e. infected any number of mosquitoes). [-] = not tested. P-value¥ = Within group comparison by Wilcoxon signed rank test (day 0 as reference). P-value† = Between ACT matched group comparison (i.e. DP with DP-PQ, PA with PA-PQ) by Wilcoxon rank-sum test. Full details of mosquito feeding assay outcomes are in Appendix pp 6 & 8. *nc* = Not calculable due to total/near total transmission reduction.

Supplementary table 4. Infectivity to mosquitoes (PA and DP arms only, ≥10 days post treatment)

A. Infectious individuals

Day of follow-up	Treatment arm	Infectious individuals (all)* % (n/N)	p-value¥	Individuals infectious (tested)* % (n/N)	p-value¥
Day 10	DP	54.17 (13/24)	0.49	54.17 (13/24)	0.49
	PA	37.50 (9/24)	0.036	37.50 (9/24)	0.036
Day 14	DP	41.67 (10/24)	0.12	41.67 (10/24)	0.12
	PA	20 (4/25)	0.0012	20 (4/25)	0.0012
Day 21	DP	21.74 (5/23)	0.0047	33.33 (5/15)	0.066
	PA	9.52 (2/21)	0.0005	25 (2/8)	0.045
Day 28	DP	4.35 (1/23)	0.0009	8.33 (1/12)	0.0084
	PA	4.55 (1/22)	0.0006	16.67 (1/6)	0.045
Day 35	DP	0 (0/23)	*nc*	0 (0/5)	*nc*
	PA	0 (0/22)	*nc*	0 (0/2)	*nc*
Day 42	DP	0 (0/23)	*nc*	0 (0/1)	*nc*
	PA	0 (0/22)	*nc*	0 (0/1)	*nc*

B. Mosquito infection (infection rate and oocyst intensity)

		i. Infectious individuals				*ii. All individuals*			
Day of follow-up	Treatment arm	Average percent infection** Median % (IQR)	p-value¥	Oocyst density Median (IQR)***	p-value¥	Average percent infection** Median % (IQR)	p-value¥	Oocyst density Median (IQR) ****	p-value¥
Day 10	DP	5.56 (0-17.74)	0.0018	0.06 (0-0.25)	0.0018	1.62 (0-11.55)	0.016	0.02 (0-0.15)	0.0091
	PA	0.74 (0-4.99)	0.030	0.01 (0-0.05)	0.034	0 (0-3.05)	0.046	0 (0-0.03)	0.046
Day 14	DP	2.27 (0-7.69)	0.0010	0.02 (0-0.08)	0.0010	0 (0-4.69)	0.0013	0 (0-0.06)	0.0013
	PA	0 (0-0)	0.0056	0 (0-0)	0.0056	0 (0-0)	0.0062	0 (0-0)	0.0057
Day 21	DP	0 (0-2.7)	0.0030	0 (0-0.03)	0.0030	0 (0-2.7)	0.0023	0 (0-0.03)	0.0023
	PA	0 (0-1.52)	0.018	0 (0-0.02)	0.018	0 (0-0.76)	0.014	0 (0-0.01)	0.014
Day 28	DP	0 (0-0)	0.0051	0 (0-0)	0.0051	0 (0-0)	0.0031	0 (0-0)	0.0031
	PA	0 (0-0)	0.043	0 (0-0)	0.043	0 (0-0)	0.035	0 (0-0)	0.035
Day 35	DP	0 (0-0)	0.043	0 (0-0)	0.043	0 (0-0)	0.043	0 (0-0)	0.043
	PA	0 (0-0)	0.18	0 (0-0)	0.18	0 (0-0)	0.18	0 (0-0)	0.18
Day 42	DP	0 (0-0)	0.32	0 (0-0)	0.32	0 (0-0)	0.32	0 (0-0)	0.32
	PA	0 (0-0)	0.32	0 (0-0)	0.32	0 (0-0)	0.32	0 (0-0)	0.32

A. Percentage of infectious individuals. *From day 7 onwards, individuals in the ACT arms (DP and PA) were only tested for infectivity in the DMFA if they were infectious to mosquitoes at one of the previous two time-points. Failure to infect mosquitoes at two subsequent time points resulted in removal from the DMFA follow-up. The number of participants infectious to mosquitoes is presented both as a total percentage of individuals still in the trial ('Infectious individuals [all]') and a percentage of the decreasing sample size being tested by DMFA as the trial progressed ('Infectious individuals [tested]'). Participants were treated with DP at day 21 of follow up to prevent re-infection and were followed-up with clinical and parasitological assessments for 49 days after treatment. Feeding was not conducted at day 49 for any participant, as no mosquito infections were observed after day 28 post-treatment. **B.** Mosquito infection measures are presented for i. all participants who were infectious at baseline, and. ii. all participants regardless of baseline infectivity. **Average percent infection = Median percentage of mosquitoes infected by each participant.. Oocyst densities are the median of all individuals' average oocyst densities. *nc* = not calculable, no positive observations. - = not tested. P-value¥ = Within group comparison. *ref* = reference group**.**

Supplementary table 5. Gametocyte circulation time and area under the curve

	Treatment group	Total gametocytes (CCP4 & PfMGET)	p-value	Female gametocytes (CCP4)	p-value	Male gametocytes (PfMGET)	p-value	p-value♂♀
Circulation time Days (95% CI)	DP	6.48 (5.31-7.65)	*ref*	5.61 (4.6-6.63)	*ref*	6.36 (5.41-7.32)	*ref*	0.29
	DP-PQ	2.07 (1.89-2.24)	<0.0001	1.99 (1.79-2.19)	<0.0001	1.81 (1.65-1.97)	<0.0001	0.16
	PA	6.46 (5.25-7.67)	*ref*	6.35 (5.01-7.69)	*ref*	5.79 (4.95-6.63)	*ref*	0.48
	PA-PQ	1.97 (1.79-2.16)	<0.0001	1.88 (1.67-2.08)	<0.0001	1.86 (1.69-2.03)	<0.0001	0.89
AUC Median (IQR) gametocytes per uL/day	DP	27.21 (18.33-50.14)	*ref*	17.64 (11.17-28.97)	*ref*	7.96 (5.63-23.36)	*ref*	0.0002
	DP-PQ	7.38 (2.16-21.62)	<0.0001	5.06 (1.52-11.76)	<0.0001	2.6 (0.74-9.15)	<0.0001	0.011
	PA	24.4 (9.92-42.02)	*ref*	14.22 (7.79-23.67)	*ref*	7.94 (2.62-15.95)	*ref*	0.0011
	PA-PQ	5.43 (2.36-19.93)	<0.0001	3.92 (1.59-12.52)	<0.0001	2.26 (0.83-5.23)	<0.0001	0.0003

Gametocyte circulation time was calculated using a deterministic compartmental model (4), and is presented as the model estimate (mean days) with 95% CI. Area under the curve (AUC) of gametocyte density per participant over time was calculated using the linear trapezoid method (5), and is presented as the median and IQR of individual AUC values by treatment arm. P-values are for differences in the t-statistic between ACT matched treatment groups. Ref = reference group. Circulation time and AUC were calculated using the first 28 days of observation only.

Supplementary table 6. Total gametocyte density, prevalence and sex ratio

	Total gametocytes (CCP4 & PfMGET)						
Day of follow-up	Treatment arm	Density Median gametocytes/μL (IQR)	p-value	Prevalence % (n/N)	p-value	Proportion male Median p (IQR)	p-value
Day 0	*Overall*	71.97 (31.01-149.53)	-	100 (96/96)	-	0.34 (0.26-0.44)	-
	DP	74.48 (37.56-126.44)	*ref*	100 (25/25)	*ref*	0.34 (0.29-0.46)	*ref*
	DP-PQ	66.15 (22.87-189.92)	0.88	100 (24/24)	*nc*	0.34 (0.25-0.44)	0.53
	PA	83.23 (34.07-123.21)	*ref*	100 (23/23)	*ref*	0.37 (0.26-0.45)	*ref*
	PA-PQ	58.95 (25.82-146.13)	0.76	100 (24/24)	*nc*	0.34 (0.21-0.44)	0.74
Day 2	DP	67.86 (33.92-119.46)	*ref*	100 (25/25)	*ref*	0.35 (0.26-0.45)	*ref*
	DP-PQ	43.06 (9.42-116.59)	0.15	100 (23/23)	*nc*	0.44 (0.24-0.63)	0.37
	PA	57.14 (27.92-102.41)	*ref*	100 (23/23)	*ref*	0.29 (0.24-0.42)	*ref*
	PA-PQ	31.34 (10.25-156.91)	0.42	100 (24/24)	*nc*	0.4 (0.22-0.48)	0.49
Day 7	DP	41.63 (16.14-68.2)	*ref*	100 (25/25)	*ref*	0.37 (0.28-0.45)	*ref*
	DP-PQ	0.1 (0-0.74)	<0.0001	69.57 (16/23)	0.0033	0.9 (0.62-0.97)	0.0036
	PA	41.38 (17.66-60.06)	*ref*	100 (24/24)	*ref*	0.35 (0.25-0.5)	*ref*
	PA-PQ	0.17 (0-1.95)	<0.0001	60.87 (14/23)	0.0006	0.51 (0.17-1)	0.25
Day 10	DP	29.83 (5.84-65.36)	-	95.65 (22/23)	-	0.36 (0.27-0.43)	-
	PA	26.66 (11.48-59.28)	-	95.83 (23/24)	-	0.31 (0.21-0.48)	-
Day 14	DP	19.17 (5.32-48.27)	*ref*	100 (23/23)	*ref*	0.31 (0.22-0.39)	*ref*
	DP-PQ	0 (0-0.08)	<0.0001	33.33 (8/24)	<0.0001	0.49 (0.49-0.49)	0.33
	PA	20.85 (9.18-33.46)	*ref*	95.83 (23/24)	*ref*	0.33 (0.2-0.48)	*ref*
	PA-PQ	0 (0-0.19)	<0.0001	43.48 (10/23)	<0.0001	0.26 (0.03-0.26)	0.45
Day 21	DP	5.42 (2.02-18.09)	*ref*	95.24 (20/21)	*ref*	0.28 (0.23-0.47)	*ref*
	DP-PQ	0 (0-0.03)	<0.0001	25 (6/24)	<0.0001	0.49 (0.49-0.49)	0.50
	PA	3.96 (2.1-8.78)	*ref*	95.24 (20/21)	*ref*	0.26 (0.18-0.44)	*ref*
	PA-PQ	0 (0-0.05)	<0.0001	36.36 (8/22)	<0.0001	0.07 (0.06-0.07)	0.035
Day 28	DP	0.9 (0.03-4.06)	*ref*	91.3 (21/23)	*ref*	0.36 (0.18-0.73)	-
	DP-PQ	0 (0-0)	<0.0001	17.39 (4/23)	<0.0001	*nc*	-
	PA	0.73 (0.08-1.5)	*ref*	90 (18/20)	*ref*	0.27 (0.06-0.36)	-
	PA-PQ	0 (0-0)	<0.0001	4.76 (1/21)	<0.0001	*nc*	-
Day 35	DP	0.14 (0-1.01)	-	73.91 (17/23)	-	0.17 (0.08-0.57)	-
	PA	0.11 (0-0.52)	-	65 (13/20)	-	0.18 (0.01-0.52)	-
Day 42	DP	0 (0-0.09)	-	26.09 (6/23)	-	0.01 (0-0.02)	-
	PA	0 (0-0.1)	-	30 (6/20)	-	0.08 (0.02-0.45)	-
Day 49	DP	0 (0-0)	-	8.7 (2/23)	-	0.02 (0.02-0.02)	-
	PA	0 (0-0)	-	9.52 (2/21)	-	0 (0-0)	-

P-values are for differences between ACT matched treatment groups. Density and proportion male were compared using Wilcoxon rank sum tests, and prevalence was compared using Fishers exact tests. For males and females, Proportion male is given for participants/time-points with total gametocyte densities of 0.2/μL and over, as described previously (3). For the calculation of gametocyte prevalence, samples were classified as negative for a particular gametocyte sex if the estimated density of in gametocytes of that sex was less than 0·01/μL (i.e. one gametocyte per 100 μL of blood sample). nc = not calculable, no observations/no observations over the threshold density for analysis. - = not tested. ref = reference group.

Supplementary table 7. Female (CCP4) and male (PfMGET) gametocyte density and prevalence

		Female gametocytes (CCP4)				Male gametocytes (PfMGET)			
Day of follow-up	Treatment arm	Density Median gametocytes/µL (IQR)	p-value	Prevalence % (n/N)	p-value	Density Median gametocytes/µL (IQR)	p-value	Prevalence % (n/N)	p-value
Day 0	*Overall*	46.55 (19.01-93.9)	-	100 (96/96)	-	22.74 (9.46-51.35)	-	100 (96/96)	-
	DP	47.14 (28.36-79.86)	*ref*	100 (25/25)	*ref*	27.33 (16.87-37.53)	*ref*	100 (25/25)	*ref*
	DP-PQ	38.91 (14.54-154.59)	1.0	100 (24/24)	*nc*	23.85 (7.68-69.07)	0.53	100 (24/24)	*nc*
	PA	45.96 (23.29-75.9)	*ref*	100 (23/23)	*ref*	21.18 (8.21-53.33)	*ref*	100 (23/23)	*ref*
	PA-PQ	44.14 (15.58-100.96)	0.87	100 (24/24)	*nc*	19.05 (9.5-50.5)	0.98	100 (24/24)	*nc*
Day 2	DP	49.6 (15.61-76.39)	*ref*	100 (25/25)	*ref*	23.65 (9.92-52.24)	*ref*	100 (25/25)	*ref*
	DP-PQ	15.71 (6.14-45.09)	0.034	100 (23/23)	*nc*	12.81 (4-38.31)	0.24	100 (23/23)	*nc*
	PA	35.42 (21.18-59.63)	*ref*	100 (23/23)	*ref*	18.07 (5.76-42.78)	*ref*	100 (23/23)	*ref*
	PA-PQ	19.97 (6.64-80.5)	0.28	100 (24/24)	*nc*	13.03 (3.21-40.71)	0.59	100 (24/24)	*nc*
Day 7	DP	22.42 (13.84-36.33)	*ref*	96 (24/25)	*ref*	13.44 (6.3-39.11)	*ref*	100 (25/25)	*ref*
	DP-PQ	0 (0-0.09)	<0.0001	39.13 (9/23)	<0.0001	0.06 (0-0.37)	<0.0001	60.87 (14/23)	0.0005
	PA	24.61 (8.54-35.31)	*ref*	100 (24/24)	*ref*	11.21 (4.12-25.41)	*ref*	100 (24/24)	*ref*
	PA-PQ	0 (0-0.59)	<0.0001	43.48 (10/23)	<0.0001	0.05 (0-0.42)	<0.0001	60.87 (14/23)	0.0006
Day 10	DP	15.61 (4.50-34.32)	-	91.3 (21/23)	-	9.26 (2.38-28.06)	-	95.65 (22/23)	-
	PA	15.61(7.57-31.65)	-	95.83 (2/-24)	-	6.94 (2.98-25.83)	-	95.83 (2/-24)	-
Day 14	DP	12.9 (4.19-30.8)	*ref*	100 (23/23)	*ref*	6.75 (2.01-21.33)	*ref*	100 (23/23)	*ref*
	DP-PQ	0 (0-0.02)	<0.0001	25 (6/24)	<0.0001	0 (0-0)	<0.0001	20.83 (5/24)	<0.0001
	PA	12.06 (7.82-18.9)	*ref*	95.83 (23/24)	*ref*	6.12 (1.88-15.16)	*ref*	95.83 (23/24)	*ref*
	PA-PQ	0 (0-0.13)	<0.0001	39.13 (9/23)	<0.0001	0 (0-0.04)	<0.0001	30.43 (7/23)	<0.0001
Day 21	DP	4.67 (1.3-11.22)	*ref*	90.48 (19/21)	*ref*	1.37 (0.5-5.09)	*ref*	90.48 (19/21)	*ref*
	DP-PQ	0 (0-0)	<0.0001	20.83 (5/24)	<0.0001	0 (0-0)	<0.0001	16.67 (4/24)	<0.0001
	PA	3.19 (1.53-5.51)	*ref*	90.48 (19/21)	*ref*	1.12 (0.63-2.85)	*ref*	90.48 (19/21)	*ref*
	PA-PQ	0 (0-0.05)	<0.0001	31.82 (7/22)	<0.0001	0 (0-0)	<0.0001	13.64 (3/22)	<0.0001
Day 28	DP	0.43 (0-2.35)	*ref*	60.87 (14/23)	*ref*	0.41 (0.03-1.05)	*ref*	86.96 (20/23)	*ref*
	DP-PQ	0 (0-0)	0.0001	13.04 (3/23)	0.0009	0 (0-0)	<0.0001	4.35 (1/23)	<0.0001
	PA	0.54 (0.04-1.29)	*ref*	80 (16/20)	*ref*	0.09 (0.01-0.39)	*ref*	75 (15/20)	*ref*
	PA-PQ	0 (0-0)	<0.0001	0 (0/21)	<0.0001	0 (0-0)	<0.0001	4.76 (1/21)	<0.0001
Day 35	DP	0.03 (0-0.69)	-	52.17 (12/23)	-	0.04 (0-0.14)	-	65.22 (15/23)	-
	PA	0.09 (0-0.29)	-	60 (12/20)	-	0 (0-0.08)	-	40 (8/20)	-
Day 42	DP	0 (0-0)	-	21.74 (5/23)	-	0 (0-0)	-	13.04 (3/23)	-
	PA	0 (0-0.07)	-	25 (5/20)	-	0 (0-0)	-	20 (4/20)	-
Day 49	DP	0 (0-0)	-	8.7 (2/23)	-	0 (0-0)	-	4.35 (1/23)	-
	PA	0 (0-0)	-	9.52 (2/21)	-	0 (0-0)	-	0 (0/21)	-

P-values are for differences between ACT matched treatment groups. Density was compared using Wilcoxon rank sum tests, and prevalence was compared using Fishers exact tests. For the calculation of gametocyte prevalence, samples were classified as negative for a particular gametocyte sex if the estimated density of in gametocytes of that sex was less than 0·01 gametocytes per µL (i.e. one gametocyte per 100 µL of blood sample). - = not tested. ref = reference group.

Supplementary figure 2. Proportion of gametocytes that were male

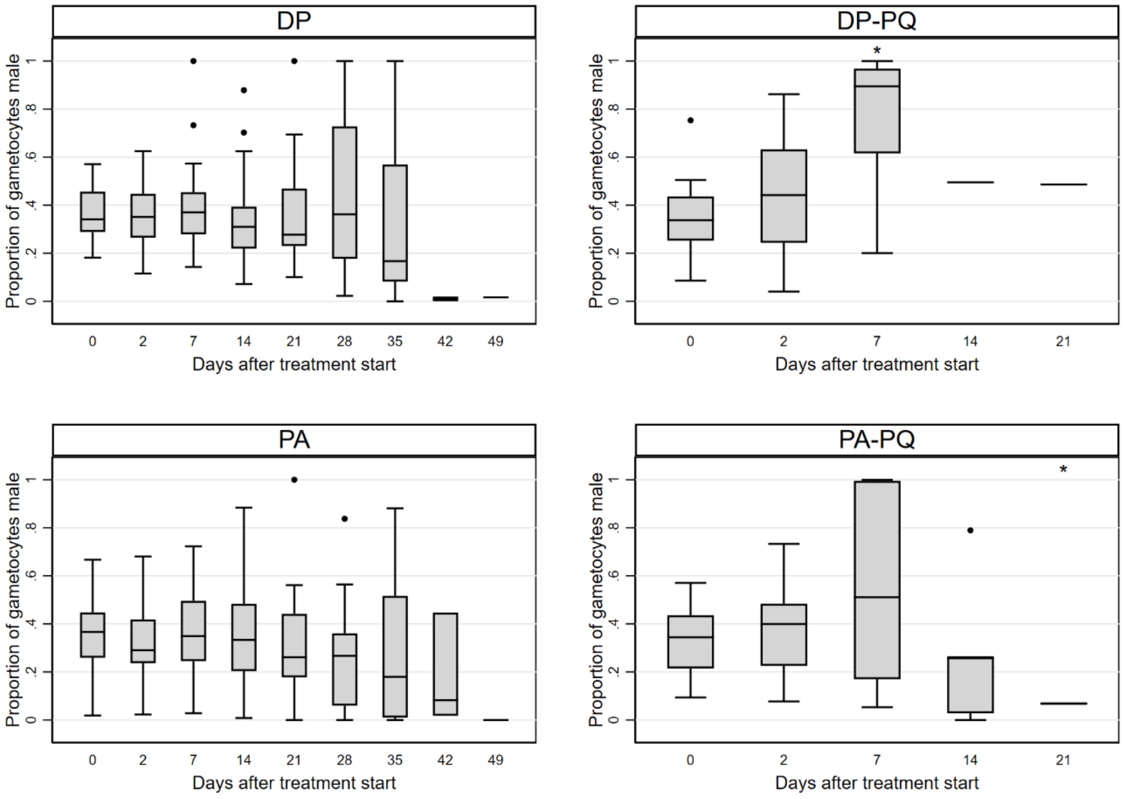

The proportion of gametocytes that were male was calculated for all values with total gametocyte densities of 0.2/µL and over, as described previously (3). P-values (<0.05) for differences between ACT matched treatment groups were calculated using Wilcoxon rank sum tests and are indicated with asterisks (*).

Supplementary table 8. Haemoglobin density and density change over time

Day of follow-up	Treatment arm	Haemoglobin density Mean g/dL (range)	p-value[¥]	p-value[†]	Percent change from day 0 Mean % (Lower/upper 95% CI)	p-value[¥]	p-value[†]
Day 0	*Overall*	12 (9.8-15.8)	-	-	-	-	-
	DP	12.1 (10-15.1)	*ref*	*ref*	*ref*	-	-
	DP-PQ	11.8 (9.9-14.4)	*ref*	0.36	*ref*	-	-
	PA	12.2 (10.2-15.8)	*ref*	*ref*	*ref*	-	-
	PA-PQ	12.1 (9.8-14.5)	*ref*	0.66	*ref*	-	-
Day 1	DP	11.6 (9.1-15.2)	0.012	*ref*	-3.88 (-6.96/-0.81)	0.016	*ref*
	DP-PQ	11.1 (9-12.9)	0.001	0.16	-6.04 (-8.52/-3.56)	<0.0001	0.26
	PA	12 (9.9-14.8)	0.099	*ref*	-2.4 (-5.47/0.66)	0.12	*ref*
	PA-PQ	11.2 (8.7-12.9)	<0.0001	0.046	-6.85 (-9.81/-3.88)	<0.0001	0.036
Day 2	DP	11.7 (9.8-15.7)	0.050	*ref*	-2.53 (-5.3/-0.24)	0.072	*ref*
	DP-PQ	11.2 (9.6-13.1)	<0.0001	0.076	-5.22 (-7.52/-2.92)	<0.0001	0.13
	PA	12.1 (9.5-16)	0.41	*ref*	-1.36 (-4.52/-1.8)	0.38	*ref*
	PA-PQ	11.6 (9.3-14.2)	0.062	0.28	-3.49 (-7.33/0.36)	0.073	0.38
Day 7	DP	11.5 (9.6-15.3)	0.021	*ref*	-4.26 (-8.11/-0.42)	0.031	*ref*
	DP-PQ	11 (8.7-12.7)	<0.0001	0.13	-6.61 (-9.33/-3.89)	<0.0001	0.31
	PA	11.7 (9.6-15.8)	0.019	*ref*	-4.02 (-7.43/-0.60)	0.023	*ref*
	PA-PQ	11.4 (9.3-13.8)	0.061	0.43	-3.77 (-8.67/-1.12)	0.12	0.93
Day 10	DP	11.71 (9.9-15.3)	0.029	-	-3.27 (-6.5/-0.03)	0.048	-
	PA	12.10 (10.3-16.1)	0.41	-	-1.28 (-4.62/2.1)	0.44	-
Day 14	DP	11.7 (10.4-16.1)	0.15	*ref*	-2.31 (-6.04/1.42)	0.21	*ref*
	DP-PQ	11.3 (9-13.3)	0.013	0.20	-4.06 (-7.43/-0.68)	0.021	0.48
	PA	12.1 (10.3-16.6)	0.35	*ref*	-1.33 (-4.28/1.61)	0.36	*ref*
	PA-PQ	11.6 (9.7-13.3)	0.13	0.22	-2.23 (-6.29/1.82)	0.27	0.71
Day 21	DP	11.9 (10.3-15.8)	0.56	*ref*	-0.5 (-4.52/3.52)	0.80	*ref*
	DP-PQ	11.7 (9.2-13.2)	0.49	0.39	-0.76 (-4.02/2.51)	0.64	0.92
	PA	12.4 (10-15.9)	0.73	*ref*	0.61 (-2.25/3.47)	0.66	*ref*
	PA-PQ	11.8 (9.6-14.1)	0.52	0.12	-0.56 (-4.27/3.14)	0.76	0.61
Day 28	DP	12 (10.3-16.5)	0.67	*ref*	-0.36 (-4.94/4.21)	0.87	*ref*
	DP-PQ	11.4 (9-13)	0.11	0.13	-2.59 (-6.27/1.09)	0.16	0.44
	PA	12.3 (9.6-15.4)	0.84	*ref*	-0.27 (-4.46/3.92)	0.90	*ref*
	PA-PQ	12.4 (10.4-19)	0.34	0.85	4.62 (-3.43/12.68)	0.25	0.27
Day 35	DP	11.9 (9.2-16.3)	0.41	*ref*	-1.18 (-5.28/2.92)	0.56	*ref*
	DP-PQ	11.8 (10-13.2)	0.87	0.88	0.79 (-2.79/4.36)	0.65	0.46
	PA	12.4 (10-16)	0.81	*ref*	0.56 (-2.83/3.95)	0.74	*ref*
	PA-PQ	11.9 (9.5-13.4)	0.65	0.13	-0.12 (-4.24/4.01)	0.95	0.80
Day 42	DP	12.2 (10.7-15.9)	0.47	*ref*	2.17 (-2.34/6.67)	0.33	*ref*
	DP-PQ	12.1 (9.5-14.5)	0.13	0.77	3.59 (-0.43/7.6)	0.078	0.63
	PA	12.7 (10.8-15.8)	0.20	*ref*	3.03 (-1.39/7.45)	0.17	*ref*
	PA-PQ	12.3 (9.6-14.8)	0.33	0.27	3.82 (-2.41/10.04)	0.22	0.83
Day 49	DP	12.4 (10.4-16.4)	0.18	*ref*	3.4 (-0.99/7.8)	0.12	*ref*
	DP-PQ	12 (8.9-13.4)	0.39	0.25	2.41 (-2.19/7)	0.29	0.75
	PA	12.8 (9.9-16.8)	0.11	*ref*	3.01 (-0.76/6.77)	0.11	*ref*
	PA-PQ	12.3 (9.4-13.6)	0.25	0.29	3.76 (-1.37/8.9)	0.14	0.81

Absolute haemoglobin density and percent change in haemoglobin density (relative to baseline) were compared within treatment arms using paired t-tests (with day 0 as reference for percent change) (p-value[¥]), and between treatment arms using two-way t-tests (p-value[†]).

Supplementary figure 3. Percent change in haemoglobin density.

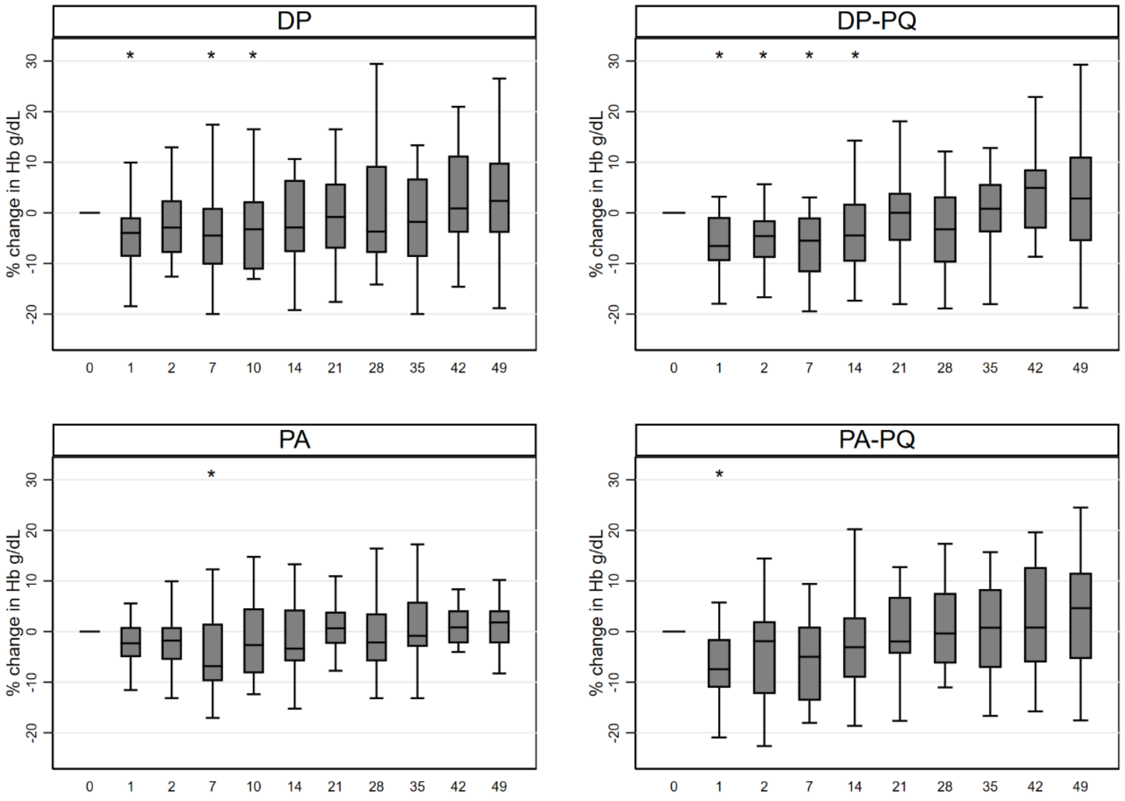

Box plots represent median within person percent change from baseline in each treatment arm. P-values (<0.05) from paired T-tests of the difference in mean percent change between time-points within each treatment arm are indicated with asterisks (*). Outliers were excluded from these plots to allow standardisation of axes.

Supplementary figure 4. Absolute haemoglobin concentration

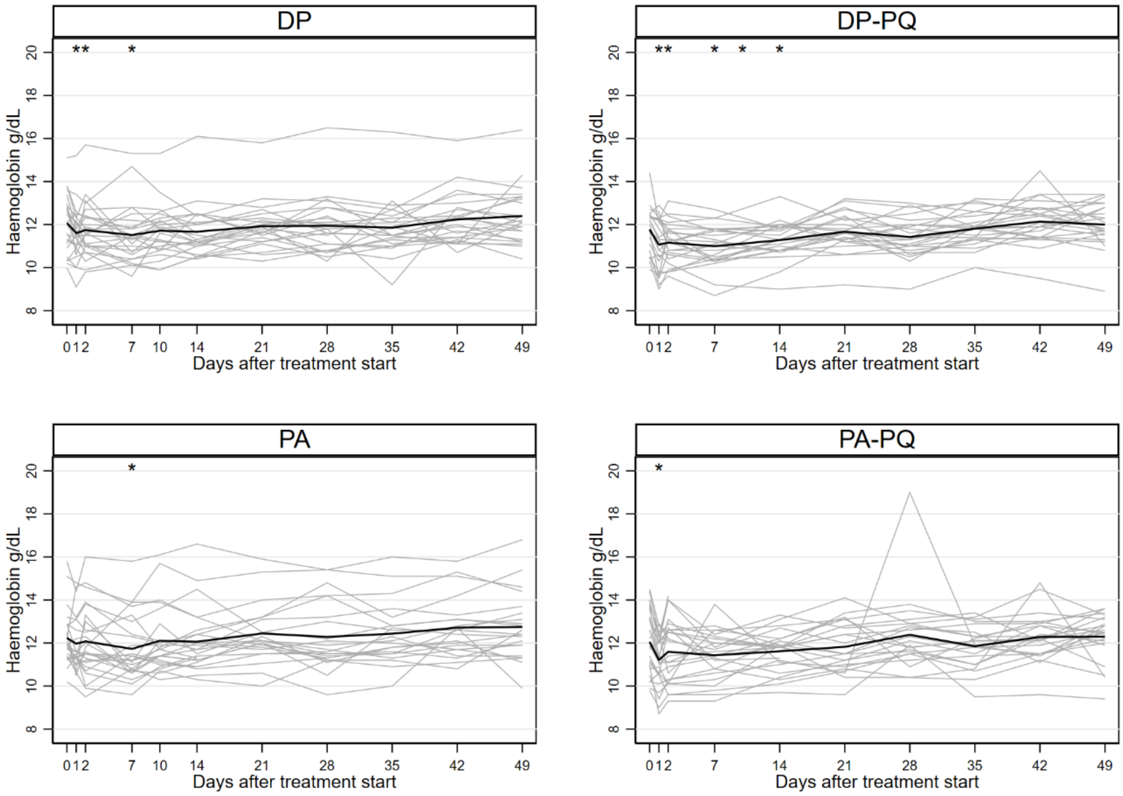

Absolute haemoglobin density is given in grams per dL (y axis, from 8-20g/dL) and is indicated for each participant individually with grey lines. The single black line shows the mean absolute haemoglobin density. P-values (<0.05) from paired t-tests for the difference in mean haemoglobin density relative to baseline (day 0) within each treatment groups are indicated with asterisks (*).

Supplementary table 9. Prevalence of adverse events

Description	All (n=100)	DP (n=25	DP-PQ (n=25)	PA (n=25)	PA-PQ (n=25)
**All**	75% (75/100)	76% (19/25)	72% (18/25)	80% (20/25)	72% (18/25)
P-value	*0.96**	*0.50***		*0.37***	
**All (mild)**	73% (73/100)	76% (19/25)	68% (17/25)	80% (20/25)	68% (17/25)
P-value	*0.75**	*0.38***		*0.26***	
**All (moderate)**	11% (11/100)	8% (2/25)	16% (4/25)	12% (3/25)	8% (2/25)
P-value	*0.90**	*0.33***		*0.50***	
**All (related to treatment)**	23% (23/100)	32% (8/25)	16% (4/25)	24% (6/25)	20% (5/25)
P-value	*0.64**	*0.16***		*0.50***	

P- values are from Fisher's exact tests for differences in proportion of individuals with an AE between all groups* or between ACT matched treatment arms**. Classification as 'related to treatment' was defined as probably, possibly or definitely related to treatment, as described in the methods.

References

1. Meerstein-Kessel L, Andolina C, Carrio E, Mahamar A, Sawa P, Diawara H, et al. A multiplex assay for the sensitive detection and quantification of male and female Plasmodium falciparum gametocytes. Malaria Journal. 2018;17(1):441.

2. Dicko A, Brown JM, Diawara H, Baber I, Mahamar A, Soumare HM, et al. Primaquine to reduce transmission of Plasmodium falciparum malaria in Mali: a single-blind, dose-ranging, adaptive randomised phase 2 trial. Lancet Infect Dis. 2016.

3. Dicko A, Roh ME, Diawara H, Mahamar A, Soumare HM, Lanke K, et al. Efficacy and safety of primaquine and methylene blue for prevention of Plasmodium falciparum transmission in Mali: a phase 2, single-blind, randomised controlled trial. Lancet Infect Dis. 2018;18(6):627-39.

4. Bousema T, Okell L, Shekalaghe S, Griffin J, Omar S, Sawa P, et al. Revisiting the circulation time of *Plasmodium falciparum* gametocytes: molecular detection methods to estimate the duration of gametocyte carriage and the effect of gametocytocidal drugs. Malaria Journal. 2010;9(1):136.

5. Méndez F, Muñoz A, Plowe CV. Use of area under the curve to characterize transmission potential after antimalarial treatment. The American Journal of Tropical Medicine and Hygiene. 2006;75(4):640-4.
